# The Prevalence and Determinants of Child Hunger and Its Associations with Early Childhood Nutritional Status among Urban Poverty Households during COVID-19 Pandemic in Petaling District, Malaysia: An Exploratory Cross-Sectional Survey

**DOI:** 10.3390/nu15102356

**Published:** 2023-05-17

**Authors:** Kai Shen Ooi, Muhammad Irfan Abdul Jalal, Jing Yuan Wong, Minn Yin Choo, Nurul Afifah Kamruldzaman, Chuan Way Lye, Lucy Chai See Lum

**Affiliations:** 1 Department of Paediatrics, University Malaya Medical Centre, Kuala Lumpur 59100, Malaysia; 2 UKM Medical Molecular Biology Institute (UMBI), UKM Medical Centre, Bandar Tun Razak, Cheras, Kuala Lumpur 56000, Malaysia; 3 Department of Public Health, University Malaya Medical Centre, Kuala Lumpur 59100, Malaysia

**Keywords:** food security, urban poor, COVID-19, dietary diversity, growth, child hunger

## Abstract

Child hunger was prevalent during the COVID-19 pandemic, but the extent, determinants, and impact on pre-school children aged 6 months to 7 years old from Malaysian urban poor households are still unknown. This exploratory cross-sectional study was performed between July 2020 and January 2021 at the Lembah Subang People Housing Project, Petaling. The households’ food security status was assessed using the previously validated Radimer/Cornell questionnaire, and the children’s anthropometric measurements were taken. Food diversity score was assessed using the World Health Organization Infant and Young Children Feeding (under-2 children) or Food and Agriculture Organization Women’s Dietary Diversity (2-year-old-and-above children) systems. Overall, 106 households were recruited. The prevalence of child hunger is 58.4% (95% CI: 50.0, 67.4). Significant differences were found in breastfeeding and sugar-sweetened beverage consumption between under-2 and ≥2-year-old children. There were no significant differences between child hunger and other food-insecure groups in weight-for-age, height-for-age, and weight-for-height z-scores. Only a higher dietary diversity score was significantly protective against child hunger after adjusting for maternal age, paternal employment status, and the number of household children (OR_adjusted_: 0.637 (95% CI: 0.443, 0.916), *p* = 0.015)). Proactive strategies are warranted to reduce child hunger during the COVID-19 pandemic by improving childhood dietary diversity.

## 1. Introduction

Food insecurity exists “whenever the accessibility to nutritionally adequate and safe foods or the ability to acquire acceptable foods in socially acceptable ways is limited or uncertain” [1]. The causes are complex and multifaceted and evolve around the four mainstays of food security: physical availability of food, economic and physical access to food, food utilization, and the stability of the aforementioned three factors [2]. Food insecurity is a multifaceted event that changes through consecutive stages of increasing severity with child hunger at the most severe level [3,4]. Hunger is defined as an uneasy or painful sensation caused by a lack of food. Its measurement captures the severity of deprivation of a basic need from resource constraints [1]. Thus, child hunger implies an extensive reduction in food intake for all household members to the extent that children also repeatedly experience hunger [3].

Food insecurity and ensuing early-life malnutrition have been linked to a multitude of adverse childhood cognitive and behavioral outcomes [4,5]. The intricate interactions between epigenetics and environmental factors have established a mechanistic relationship between nutrition, genetics, and illness. Nutrition-sensitive epigenomic disruption has been shown to have long-lasting detrimental effects on neurodevelopment, immune function, and liver health [6,7]. Shankar et al. observed that food insecurity has deleterious impact on the social, behavioral, and emotional aspects of four distinct groups of children—toddlers, pre-school children, school-aged children, and adolescents [4]. Furthermore, Simonovich et al. demonstrated that food insecurity might lead to obesity, anemia, increased cortisol, and low birth weight [8] in under-5 children. These observations may be associated with the households’ coping strategies to deal with the inherent community-wide poverty or loss of income and employment, which were secondary effects of the economic shock caused by the coronavirus (COVID-19) pandemic [9,10].

Despite Malaysia being nationally considered food-secure, the food insecurity that prevails among low-income households jeopardizes the dietary quality and sufficiency of macro- and micronutrient intake [11]. The widening socioeconomic gap has caused the government to initiate efforts to reduce urban poverty among the Bottom 40% (B40) group, defined as households earning 40% or below the national monthly household income distribution [12]. UNICEF reported that 12% of children living in the low-cost flats of Kuala Lumpur were food insecure, with less than three meals a day, and 97% of all households surveyed mentioned that high food prices and insufficient income prevented them from preparing healthy meals for their children [13]. Among children under five years of age, 15% were underweight, 22% stunted, 20% wasted, and 23% overweight or obese. These data are consistent with the Malaysia National Health and Morbidity Survey (NHMS) 2019 [14]. Furthermore, we found a high proportion of severe wasting in our recently published small pilot randomized controlled trial involving under-5 children from the B40 households [15]. This underscores the need for additional investigations on the factors associated with poor childhood nutrition and child hunger in this economically deprived community, especially during global public health emergencies such as the nationwide lockdown era during the COVID-19 pandemic.

During the COVID-19 pandemic, Malaysia’s rank in the Global Food Security Index (GFSI) dropped from 33rd in 2019 to 43rd in 2020 based on the adjusted overall GFSI score [16,17]. Nevertheless, the economic turmoil from the COVID-19 pandemic caused negative economic growth from the prolonged lockdowns, resulting in enormous public health and social safety implications [18]. The Department of Statistics Malaysia (2020) reported that most employees were unprepared for the extended lockdown; approximately three-quarters (71.4%) of self-employed people had sufficient savings for less than one month, whilst the financial sufficiency among employers (77.2%) and private employees (82.7%) could barely last up to 2 months [19]. Compounding the problem further, less than half of the self-employed employees (46.6%) lost their jobs, and almost all self-employed workers (94.8%) experienced substantial reductions in monthly income—which could be up to 90% of their total monthly earnings in more than a third (35.5%) of cases—during the COVID-19 pandemic [19]. This shows the extent of financial fragility among the Malaysian workforce, especially those from the B40 households.

Heretofore, there has been a scarcity of studies looking into the negative impact of the COVID-19 pandemic on early childhood nutrition and childhood hunger among households experiencing urban poverty in Petaling district, the most populous administrative district in Malaysia [20,21,22,23,24,25]. Hence, our study aims are three-fold: (i) to establish the prevalence of child hunger among an urban poor B40 community in Petaling during the COVID-19 pandemic; (ii) to assess the impact of child hunger on children’s anthropometric measurements and its associations with dietary intake and nutritional status; and (iii) to determine the risk factors of child hunger, the most severe form of food insecurity, among the urban poor B40 households during the COVID-19 pandemic.

## 2. Materials and Methods

### 2.1. Study Design

This is a single-site cross-sectional study involving children aged between 6 months and below 7 years (pre-school children) living in the People’s Housing Project (*Program Perumahan Rakyat* (PPR)) Lembah Subang 1, Malaysia, from July 2020 until January 2021. This research was reported according to the Strengthening of the Reporting of Observational Studies in Epidemiology (STROBE) and the Consensus-Based Checklist for Reporting of Survey Studies (CROSS) guidelines since this research employed a questionnaire-based survey tool for capturing the information on study endpoints [26,27].

### 2.2. Inclusion and Exclusion Criteria

All children aged between 6 months and 7 years old (pre-school children) living in the People’s Housing Project (*Program Perumahan Rakyat* (PPR)) Lembah Subang 1 were included in this study. Syndromic children and those with severe chronic illnesses were excluded from the study.

### 2.3. Study Setting, Participant Recruitment, and Sample Representativeness

The target community for this study was the residents of the low-income households at the People’s Housing Project (also known as PPR) Lembah Subang 1, located in Central Ara Damansara, Petaling Jaya, Selangor, Malaysia. Moreover, known as PPR Taman Putra Damai, this People’s Housing Project is part of a nationwide initiative by the Malaysia Government to provide affordable housing for the urban poor. The estimated population of the locality is more than 15,000 residents. The PPR Lembah Subang 1 is the largest PPR in Petaling district.

The on-site recruitment occurred between July 2020 and January 2021. However, the community recruitment process was disrupted during the nationwide Movement Control Order (MCO), a form of cordon sanitaire imposed by the Malaysia Government. For each household, only one child (the youngest child) was selected to eliminate the intra-household correlation and hierarchical (multi-level) structure of the data that may complicate the statistical analysis.

A purposive sampling method was employed to select the participants due to the difficulties in accessing the entire sampling frame caused by the strict MCO, a common issue experienced by many studies carried out in Malaysia during the COVID-19-related lockdown period [28,29]. Theoretically, this is justified by a number of prior studies which showed the acceptability of a purposive sampling approach in sampling marginalized populations [30,31]. Moreover, a purposive sampling scheme is also appropriate for exploratory research or piloting purposes [32]. Furthermore, the sampling area (PPR Lembah Subang 1) covers a vast number of B40 households in the whole administrative district of Petaling, signifying the appropriateness of our sampling site.

### 2.4. Ethical Issues

The protocol for this study was approved by the University Malaya Medical Centre Medical Research Ethics Committee (MREC ID no: 2020410-8500). The study was conducted in line with the Helsinki Declaration [33]. Written informed consent was obtained from each caregiver (parents or legal guardians of the children) prior to study enrolment. No participants did received any form of financial incentive for participating in this study.

### 2.5. Sample Size Calculation

The sample size required for determining the prevalence of child hunger was calculated using a single proportion formula [34] as below:

(1) $$n={\left(\frac{Z_{\alpha /2}}{d}\right)}^{2}\times p\;\left(1-p\right),$$

where *n* is the calculated sample size; *z_α_*_/2_ is the value of standard normal *z*-score at 95% confidence level, which is 1.96; *d* is the margin of error (or precision), which we assumed to be 8.5% in our case; and *p* is the prevalence of child hunger from previous research. The prevalence of child hunger in Malaysia was estimated to be between 15.0–40.8% among low-income households located in deprived areas (except urban-poor (B40) households) [11]. For our sample size calculation, we assumed the midpoint of such a range of estimates (27.9%) as the prevalence of child hunger in our setting. The sample size required for this research objective is thus 107 households.

For the second research objective, the required sample size was calculated using PS Software Version 3.1.2 [35]. The type I (α) and power of the study (1-β) were fixed at 0.05 and 0.80, respectively. The case-to-control ratio (m) was fixed at a 1:1 ratio; P_1_ represents the proportion of children experiencing child hunger having the risk factors; and P_0_ is the proportion of children without child hunger having the risk factors. We also assumed a 10% attrition rate to account for missing data and inaccurate recordings of quantitative outcome variables such as the weight and height of the participants. Only one prior research article [36] provided the relevant estimates for sample size calculation, and they were only available for 4 risk factors (mother’s education, father’s education, single-parent households, and receiving financial aid status) of child hunger. Moreover, we also assumed that the minimum detectable effects (MDEs) (P_1_ − P_0_) for each determinant are slightly bigger than those reported in McIntyre, Connor, and Warren [36] due to the nature of our population; it is comprised of households experiencing urban poverty. The calculated sample size for each risk factor is given in Table 1 below.

Hence, the overall calculated sample size required for our study is 107 households.

### 2.6. Data Collection and Study Instruments

Strict standard operating procedure was adhered to per University Malaya guidelines for fieldwork and research activities during the Recovery Movement Control Order. Approval for fieldwork was given by University Malaya and the Royal Malaysia Police. A total of 13 field trips were made to the community, and about 20 participants were recruited during each trip. Four stations were allocated for registration and COVID-19 screening, face-to-face interviews for written consent and structured questionnaire administration, and growth parameters measurement.

#### 2.6.1. A Structured Questionnaire

A structured questionnaire consisting of separate sections on the household’s socioeconomic and demographic characteristics, dietary assessment based the on 24-h dietary recall method, and food insecurity status was administered through a face-to-face interview by the research assistants, who had been trained in survey data collection processes based on the previously described method [15]. The interview required about 15 to 20 min to complete. A sample of the used questionnaire is provided in Files S1–S7 (Supplementary Material).

#### 2.6.2. Anthropometric Measurements of the Household Children

The anthropometric measurements were measured with the children in light clothing and no shoes using Portable SECA 874 mobile flat scales (SECA, Hanover, MD, USA), which are accurate to 0.1 kg. The height was measured in recumbent length for children up to 2 years old using a SECA 417 mobile measuring board (SECA, Hanover, MD, USA), whereas for children 2–5 years old, the height was measured using a SECA 217 stadiometer (SECA, Hanover, MD, USA), which is accurate to the nearest 0.1 cm. All equipment was calibrated twice weekly. The same procedures were implemented when measuring parental height and weight.

Measurements were conducted by the pediatricians, with the additional assistance of medical student volunteers from University Malaya. Birth weights were obtained from the children’s growth and immunization record book issued by the Ministry of Health of Malaysia. All children’s anthropometric data were converted to the age-standardized z-scores based on the WHO guideline, including weight-for-age z-score, height-for-age z-score, and weight-for-height z-score [37].

#### 2.6.3. Operational Definitions of Dietary Intake, Minimum Dietary Diversity (MDD), and Sugar-Sweetened Beverages

The information on dietary intake was obtained using the 24-h food recall questionnaire adapted from the WHO Infant and Young Child Feeding (WHO-IYCF), Kennedy et al., and Martin-Prével et al. guidelines [38,39,40]. Parents were further asked about their children’s consumption of a list of local foods in various food groups (FGs).

For children aged 6 to 23 months, the dietary diversity score (DDS), a surrogate barometer of nutrient sufficiency, was based on 8 FGs [38]. The operational definition of food intake adequacy for each FG was slightly modified from the updated 2017 WHO-IYCF guideline and thus defined as the consumption of any quantity of any type of food or beverage within such FG [38]. The accuracy of our utilized operational definition is further corroborated by the latest 2021 WHO-IYCF guideline published after our study’s conclusion [41]. A cut-off value of 5 FGs and more in 24 h was taken as the MDD score [38].

For pre-school children aged 24 months and above, dietary diversity was based on the Women’s Dietary Diversity (WDD) scale, which comprises 9 FGs, as recommended by Kennedy et al. and Martin-Prével et al. [39,40]. It was originally developed for women aged between 15 to 49 years old (reproductive age group), but the scale could be used for individuals from different age groups and gender [40]. For each FG, a satisfactory intake (score 1) is defined as consuming at least 15 g of such food group within the previous 24 h [40], while an unsatisfactory intake was given a score of 0. A threshold of at least 5 out of 9 FGs defined MDD for children within this age group [39].

Sugar-sweetened beverages (SSB) are defined as all types of drinks that contain free sugar, such as monosaccharides (e.g., glucose, fructose) or disaccharides (sucrose or table sugar), and these include carbonated and non-carbonated soft drinks, whole fruit/vegetable juices or drinks, ready-to-drink coffee or tea, energy sports drinks, flavored water, or flavored milk drink [42,43].

#### 2.6.4. Assessment of Food Insecurity and Child Hunger

The Radimer/Cornell Hunger and Food Insecurity Instrument was used to assess the food security status in the household. The instrument had been translated into Malay and validated by Sharif et al. [44]. The translated questionnaire has a good-to-excellent internal consistency (Cronbach’s alpha: 0.91 (household hunger); 0.89 (child hunger); and 0.92 (individual hunger)) and satisfactory construct validity (Pearson’s r for correlations between each construct to the items: 0.20–0.75 (household hunger); 0.32–0.73 (child hunger); and 0.36–0.76 (individual hunger)) [45].

The questionnaire consists of 10 items derived from 4 different conceptual components of hunger: quantity of food, quality of food, psychological effects of food deprivation, and social implications due to disrupted eating patterns and food procurement [46,47]. These items could be answered as “not true”, “sometimes true”, or “often true”, and the answers were further dichotomized into negative (“not true”) and positive (“sometimes true” or “often true”) answers. Based on the respondent’s answers, food insecurity can be classified into four categories with increasing severity—food-secure (negative answers to all items), household food-insecure (positive answers to one or more items of item 1 to 4), individual/adult food-insecure (positive answers to one or more of item 5 to 8), and child hunger (positive answer to items 9 and 10) [45].

### 2.7. Statistical Analysis

The sociodemographics characteristics of the respondents, anthropometric measurements, COVID-19 relief aid, and the dietary intake of the children were descriptively summarized as mean (standard deviation, SD) or frequency (percentage) for continuous and categorical variables, respectively. For non-normal continuous variables, median and interquartile range (IQR) were reported instead. Multiple imputation with multivariate imputation using chain equation (MICE) technique [48] would be carried out under the missing at random (MAR) framework if the percentage of missing data was between >5% and <40% for each variable [49]. Otherwise, only complete case analysis was performed since multiple imputation is not recommended if the percentage of missing data is small (<5%) or excessively large (>40%) [49]. The normality of continuous covariates was assessed using the Shapiro-Wilk test, histograms, box-and-whisker and QQ plots and Fisher’s skewness coefficient (threshold ±1.96). The homogeneity of variances of the continuous outcome variables between the child hunger and the combined household and individual food insecure groups was examined using Levene’s test.

The associations between child hunger and children’s and parental anthropometric measurements, children’s nutritional status, and dietary intake were assessed using the exact version of Chi-Square test (Mehta and Nitin algorithm) [50] for categorical covariates or the independent *t*-test if the continuous covariates are normally distributed. Otherwise, the Mann–Whitney test was used for non-normally distributed covariates.

For height-for-age and weight-for-height z-scores, multi-way ANOVA was performed. Potential confounders, such as breastfeeding status and SSB, were assessed for significance first and then included into the multi-way ANOVA model containing the child hunger variable if they are significant. Furthermore, for weight-for-age z-score, analysis of covariance (ANCOVA) was performed where the birthweight was treated as the model covariate and food insecurity groups (combined household and individual insecure versus child hunger) and child’s age group (toddlers aged <2 years old versus pre-school children aged ≥2 years old) were used as the model factors. The two-way ANOVA and ANCOVA assumptions, such as linearity, independence, normality, and heterogeneity of residual variances, were visually inspected using residual versus predicted value plot. The homogeneity of regression line assumption for ANCOVA was examined by evaluating the statistical significance of the factor x covariate interaction term.

A simple logistic regression was carried out to screen for the potential determinants of child hunger. Variables with a *p*-value of less than 0.25 or clinically and theoretically important variables were chosen for the multivariable model building using multiple logistic regression [51]. For predictors with sparse data structure, the Firth logistic regression procedure was utilized [52,53]. Forward and backward stepwise methods with the default p_Entry_ = 0.05 and p_removal_ = 0.10 were used to build a multiple logistic regression model for the association between the potential determinants and child hunger with multivariable adjustments made for the effects of theoretically well-known determinants (confounders) of child hunger such as maternal age, number of children in the household, and paternal employment status [36,54,55], regardless of their statistical significance at the univariable level [51,56,57].

The *p*-values based on the Wald Statistic (for continuous or dichotomous categorical predictors) or partial likelihood ratio test (for categorical predictors with more than 2 levels of categories) were utilized to assess the significance of the predictors (determinants) of child hunger for inclusion into the multivariable model [51]. The presence of plausible effect modifiers was checked by examining the statistical significance of the interactions between the variables included in the multiple logistic regression model. Multicollinearity between predictors was checked using these methods and thresholds: inspection of the correlation matrix (inter-predictor correlation ≥ 0.70), variance inflation factor (VIF ≥ 4), tolerance (≤0.25), and condition indices (≥30) [51,58].

The model fit and discriminatory properties were then assessed using the Hosmer–Lemeshow test, percentage of correct classification and area under the receiver operating characteristic (AUC) curve (threshold of satisfactory discrimination: 70% or above) [51]. The presence of influential observations was examined using dFBeta (threshold: 1) and leverage (critical value: 2p/n; where p is the number of the predictor including the model’s intercept). They would be removed if the values were theoretically implausible and retained if the values were theoretically correct [51,59]. The final model equation was presented for predictive purposes, and the results were presented as crude and adjusted odds ratios (ORadj) together with their 95% confidence interval (95% CI). No sensitivity analysis was performed since this was not planned a priori.

All statistical analyses were performed using the IBM Statistical Package for Social Science (SPSS), version 26 (IBM Corp., Armonk, NY, USA), and STATA, version 15.1 (StataCorp., College Station, TX, USA). The *p*-value < 0.05 was selected as the significance threshold, and all statistical tests were two-tailed procedures.

## 3. Results

### 3.1. Patient Recruitment

**Figure 1:**
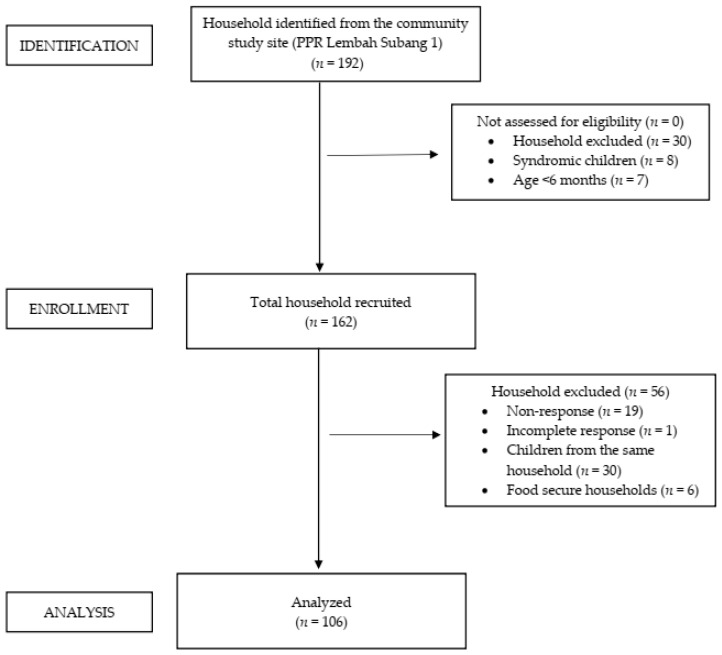
STROBE flow diagram for a cross-sectional study, detailing the progress of each study phase.

Initially, 192 households were screened and assessed for study eligibility during the early recruitment phase. Of these, 30 households were excluded due to the child’s chronic illness (*n* = 15) and clinical syndrome (*n* = 8). A further seven households with children less than 6 months old were removed. In total, 162 households were successfully enrolled on recruitment; however, an additional 56 households were omitted from the study due to non-response (*n* = 19), incomplete questionnaire response (*n* = 1), food secure household (*n* = 6), and children from the same household (*n* = 30). In the end, only 106 households were included in the data analysis. The progress of each phase of this study is summarized in Figure 1 below.

### 3.2. Clinicodemographic Profile of the Households

A total of 112 households were enrolled. Of these, 106 (94.6% (95% CI: 88.8, 97.5) households experienced food insecurity. Only six households had food security and were thus excluded from the analysis. The food insecure households were categorized into three mutually exclusive groups of increasing severity of food insecurity: 20 (18.9% (95% CI: 12.6, 27.3)) reporting household food insecurity; 24 (22.6% (95% CI: 15.7, 31.5)) individual food insecurity; and 62 (58.4% (95% CI: 50.0, 67.4)) with child hunger. Considering the relatively small sample size, both household and individual food insecure groups were combined for data analysis. The associations between the two groups (combined household and individual insecure group and child hunger group) and the baseline clinical and sociodemographic characteristics are presented in Table 2. 

From Table 2, out of 106 households, there were 3 families without fathers (single-parent families). No significant differences were found between the two food insecure groups, i.e., combined household and individual insecure and child hunger groups, regarding the clinical/sociodemographic variables. Overall, 98 out of 106 (92.5%) households had a monthly income of less than RM 3000. Slightly more households in the child hunger groups received financial and food assistance and had lower total household incomes than the combined household and individual insecure groups. More fathers were employed compared to mothers, and most parents attained secondary education as their highest education level. Based on Table S1 (Supplementary Material) results, most households received staple food, cooking oil, and sugar as their COVID-19 food aid relief.

### 3.3. Differential Impact of Food Insecurity Levels on Children’s and Parental Anthropometric Measurements

The significance of the confounders—breastfeeding status and SSB consumption—were assessed first. Based on the results presented in Table 3, the proportions of breastfeeding status and SSB consumption were significantly different in pre-school children aged 2 and above and toddlers under the age of 2 years old. These were then selected and treated as confounders.

From Table 4, no significant differences and associations were observed between the two food insecure groups regarding children’s and parental anthropometric measurements after adjusting for the confounders for the weight-for-age z-score, height-for-age z-score, and weight-for-height z-score endpoints (please refer to the footnotes of Table 4). After controlling for the effects of breastfeeding status and SSB consumption, the association of child hunger with both stunting (ORadj: 0.482 (95% CI: 0.162, 1.438); *p* = 0.191) and wasting (ORadj: 1.386 (95% CI: 0.532, 3.607); *p* = 0.504) were not significant.

### 3.4. Associations between Dietary Diversity and Child Hunger

From Table 5, the median DDS was significantly higher in the combined household and individual insecure group than in the child hunger group for the under-2 children. Although the combined household and individual insecure group had higher proportions of intake for the majority of the food groups, it is the DDS, rather than the specific food groups, that had a significant association with child hunger for the under-2 toddlers. On the contrary, in children aged 2 years and above, neither DDS nor food groups were significantly associated with food security levels. For SSB, no significant difference was found between the two food insecurity groups when the observations from both age groups were combined (χ^2^ statistics (df): 0.820 (1), exact *p*-value = 0.426).

### 3.5. Sociodemographic and Dietary Determinants of Child Hunger

Only 103 households were analyzed for this analysis since 3 families (2.83%) were single-parent with missing values for paternal employment and highest educational status. Based on simple logistic regression analysis, we found three variables that reached the preliminary significant threshold of *p* = 0.25: total household income; financial aid and the dietary diversity score (DDS). Based on the forward and backward stepwise variable selection procedures, our multiple logistic regression analysis revealed that DDS was the sole significant predictor of child hunger after controlling for the effects of maternal age, paternal employment status, and the number of children in the households. Moreover, based on a purposeful selection of predictors in the model, no predictor was found to have achieved statistical significance. The full results are given in Table 6.

In summary, while the sociodemographic characteristics, anthropometric measurements, and consumption of individual FGs between the two food insecure groups were not significantly different, it emerged that the DDS is the only significant determinant of child hunger. We found that a unit increase in DDS would reduce the odds of child hunger by 36.3%. The logit equation that describes the relationship between child hunger and its predictors is given by Equation (2):

(2) $$\mathrm{logit}\;(\frac{\mathrm{Pr}\;\left(\mathrm{child}\;\mathrm{hunger}\right)}{1-\;\mathrm{Pr}\;\left(\mathrm{child}\;\mathrm{hunger}\right)})=\;4.088-0.451\;*\;(\mathrm{DDS})-0.047\;*\;(\mathrm{Mother}’s\;\mathrm{age})-0.532\;(\mathrm{paternal}\;\mathrm{employment})+0.064\;(\mathrm{no}\;\mathrm{of}\;\mathrm{children})$$

Thus, the probability of child hunger (Pr (child hunger)) is given by Equation (3):

(3) $$\mathrm{Pr}\;(\mathrm{child}\;\mathrm{hunger})=\;\frac{e^{4.088-0.451\;*\;\left(\mathrm{DDS}\right)-0.047\;*\;\left(\mathrm{mother}’s\;\mathrm{age}\right)-0.532\left(\mathrm{paternal}\;\mathrm{employment}\right)+0.064\;*\;\left(\mathrm{number}\;\mathrm{of}\;\mathrm{children}\right)}\;}{1+e^{4.088-0.451\;*\;\left(\mathrm{DDS}\right)-0.047\;*\;\left(\mathrm{mother}’s\;\mathrm{age}\right)-0.532\;\left(\mathrm{paternal}\;\mathrm{emplyment}\right)+0.064\;\left(\mathrm{number}\;\mathrm{of}\;\mathrm{children}\right)}}\;,$$

where *e* is an exponential constant (e.g., *e*^1^ = 2.718) and ∗ is a multiplication symbol.

## 4. Discussion

### 4.1. The Prevalence of Food Insecurity and Child Hunger

Food insecurity and child hunger are known to be exacerbated by the sudden occurrence of public health emergencies, such as the COVID-19 pandemic, that create worldwide economic shock and disrupt the global food supply chain [60]. Our study found a high prevalence of food insecurity (95.4%) and child hunger (58.4%) among the B40 community living in a People’s Housing Project in the Petaling administrative district during the COVID-19-related MCO period. These rates are higher than those prior to the COVID-19 pandemic, where food insecurity was 48.2–85.2% and child hunger 15.0–40.8% among low-income or welfare beneficiary households [11], and those reported by UNICEF study that covered the low-cost flats of urban Selangor [13]. Our estimates are also higher than those reported by Rengarajoo and Tan (food insecurity: 67.6%; child hunger: 29.7%) [61]. The inconsistencies can be explained by their different study inclusion criteria (parents with healthy children aged 2–11 years in Malaysia) and data collection method (Google Form-based survey questionnaire through social media platforms) which might increase the response error rate [62,63,64,65], thus causing them to underestimate the true prevalence of food insecurity among the low-income households during the MCO period [61]. Due to the strength of our data collection process (i.e., face-to-face interviews), we believe our findings reflect the extent of food insecurity and child hunger among the B40 households in the urban Petaling administrative district more precisely than what the previous research had documented. We also noted that the prevalence of households experiencing child hunger is much higher than both the household and individual food insecure groups—an expected result since parents would relinquish their nutritional requirements to protect their children’s nutritional demands [9].

Surprisingly, the prevalence rates of B40 households experiencing food insecurity and child hunger are also higher than those who might experience more severe economic hardships during the COVID-19 pandemic, such as the Palestinians in the conflict region of Gaza (food insecurity: 71.5%; child hunger: 21.5%) [66] and the two urban slums of Dhaka, Bangladesh (food insecurity: 21.5% child hunger: 1.4%) [67]. This unexpected discrepancy could be attributed to the more severe pre-pandemic prevalence of food insecurity among the urban poor and more prolonged and stricter MCO in our local setting resulting in a high level of joblessness and significant reductions in monthly earnings. About 92% of households in our study earned less than RM 3000 (approximately USD 690) per month during the MCO period, well below the median household income of the B40 group [68], indicating the pervasiveness of urban poverty. Our findings may encourage the relevant stakeholders (e.g., national policymakers, non-governmental organizations) to address this issue more proactively since low-income households are less resilient to economic rebound to the pre-pandemic level after the country’s economic reopening [69].

### 4.2. Anthropometric Measurements and Child Hunger

In this study, more than a sixth (16.0%) and nearly a quarter (22.6%) of children were stunted or wasted, respectively. Compared to NHMS 2019 estimates of 21.7% (95% CI: 17.35, 26.72) and 10.1% (95% CI: 7.47, 13.43) for stunting and wasting, respectively, for under-5 children living in the urban area [61], our estimates were lower for stunting and higher for wasting. These discrepancies might be explained by our study including children between 5 to 7 years old, an age group reported by NHMS 2019 to show a lower rate of stunting in urban areas (11.3% (95% CI: 9.36, 13.47)) [14].

Furthermore, no differences were found in growth parameters between our two groups of food insecurity, which can be attributed to the adequate food and financial relief received by the households during the MCO period. Our findings are thus in conformity with the observations made by Nepali et al., who showed no significant association (*p* = 0.587, based on the reanalysis of the author’s data using exact χ^2^ test and with the food secure group removed) between the increasing level of food insecurity and wasting among under-5 children in Nepal [70]. Moreover, our finding is also corroborated by Pathak and colleagues, who demonstrated a non-significant association (*p* = 0.657, based on the reanalysis of the author’s data using exact χ^2^ test and with the food secure group removed and the moderate and severe stunting categories combined) between food insecurity level and stunting among under-5 children in Dibrugarh, Assam [71].

We hypothesized that the possible shorter duration of the severe nutritional deficiency state experienced by the children in our sample had prevented a sufficient number of occurrences of wasted and stunted cases in both combined household and individual insecure and child hunger groups, which thus affected our study’s power to detect any significant difference in anthropometric endpoint between those two groups. Moreover, it is also possible that parents adopted sufficient coping strategies, such as restricting their dietary quality and quantity so that their children could have a satisfactory quantity and quality of dietary intake [44].

### 4.3. Dietary Diversity Score (DDS) as the Determinant of Child Hunger among the Low-Income PPR Households

A significant association between child hunger and DDS was demonstrated in children under the age of 2 among the B40 in low-cost flat populations in the Petaling administrative district, but not in those aged 2 and above. A similar association between food insecurity and/or child hunger and DDS has been observed in different types of urban-poor communities [72,73]. In contrast to Ali et al., we demonstrated differential age-group effects of DDS on child hunger; this could be attributed to the higher proportion of breastfed children in the combined household and individual food-insecure groups. Moreover, the differences in the proportions of children consuming each food group between the combined household and food insecure and child hunger groups are much higher in children under 2 years old than those aged two years and above. Based on this, we deduce that children’s dietary diversity intake carries a more influential role in preventing child hunger than the intake of specific food groups, and these effects are more prominently observed in under-2 children. The closure of schools during the MCO period also limited the children’s access to the Supplementary Food Program (*Rancangan Makanan Tambahan*) provided by the Ministry of Education, Malaysia, further reducing their access to food and balanced nutrition. This might also be another reason for an increase in the prevalence of child hunger in our sample.

### 4.4. The Statistical Endogeneity Issue between Dietary Diversity Score (DDS) and Child Hunger

We also realized the significance of DDS as a child hunger determinant may also be confounded by other potential social determinants such as total household income, parental employment status and highest education levels, financial aid status, and total number of children within the households. This endogeneity issue between DDS and child hunger or child nutritional status, particularly, is more important in social science and econometrics [74,75] than in nutritional sciences [76], and ignoring this problem may introduce significant systematic bias to the estimates of the regression coefficient for DDS, as presented in Table 6. To address this issue, we performed additional analyses where we treated DDS as an endogenous (dependent) variable and regressed it on the other potential social determinants of child hunger.

**Figure 2:**
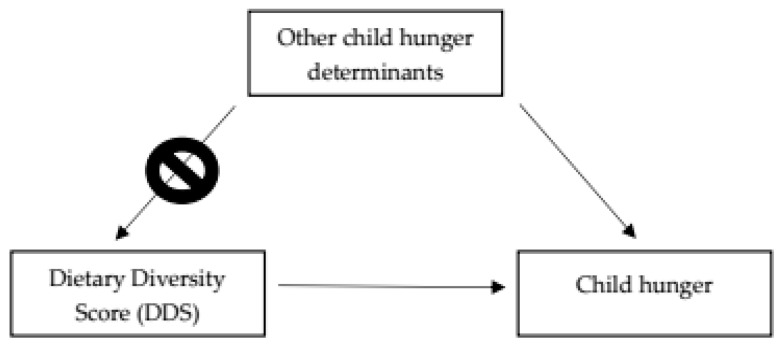
The directed acyclic graph (DAG) representing the causal relationships between other child hunger determinants, DDS, and child hunger. The DDS <- Other child hunger determinants -> path is the blocked, non-causal, spurious backdoor pathway between DDS and child hunger due to the independence of DDS and other child hunger determinants.

From the results in Supplementary Table S2, we did not find any significant associations between all other potential child hunger determinants and DDS. Hence, within this population of food insecure households living in an urban-poor community, other social determinants of child hunger are independent of DDS. Based on the path (causal) diagram below (Figure 2), we conclude that the spurious backdoor pathway (DDS <- other potential child hunger determinants -> child hunger) is blocked. Therefore, we consider the estimated effect of DDS on child hunger in this population of household food insecure families to be systematically unbiased and trustworthy.

### 4.5. Strengths and Limitations

Our study is the first to investigate the prevalence of food insecurity and child hunger among the urban poor community during the height of the COVID-19 pandemic. Our results could thus be utilized to prevent the worsening of food insecurity and child hunger among urban poor households, especially during public health emergencies, through the formulation of meticulous mitigation strategies. Furthermore, we also employed a face-to-face data collection process which reduces the non-response error rate and the possibility of recall bias. In addition, our study also addressed a major issue that was and is still pervasive among the urban poor families during a time of uncertain economic situation in the country.

In light of some study limitations, the results must be interpreted cautiously. First, the cross-sectional design employed by our study may prevent the determination of a causal link between DDS and child hunger due to the absence of a temporal relationship between cause and effect. This study only reflects the current food insecurity and nutritional status of children of poor households during the COVID-19 pandemic lockdown. It may not represent the situation at the pre- or post-pandemic time frame. Second, the generalizability of our study findings to other B40 households in the Petaling administrative district is fairly limited in view of the small sample size, selection bias, and issues with sample representativeness secondary to the non-probability purposive sampling scheme employed and the limited sampling site (a single PPR). This was caused by (i) a large number of potential study participants who did not respond to our study invitations; (ii) that a significant number of them returned to their villages due to the MCO-related loss of livelihood; and (iii) that many of them declined to participate due to fear of contracting COVID-19. This inevitable shortcoming impacted on our original plan to carry out the sampling of participants via a stratified random sampling scheme using housing blocks as our stratification variable. Nevertheless, we are in the process of confirming our exploratory findings in our current ongoing study, which will be completed by the end of 2023. Third, the differential impacts of increasing levels of food insecurity could not be assessed due to the limited number of households experiencing household and individual insecurity, warranting the combination of both groups as the reference group. Fourth, this study only evaluated the impact of food insecurity quantitatively, whilst ignoring the qualitative impact of food insecurity and child hunger, such as the household’s specific and personal perception, experiences, and coping strategies used to mitigate the effects of food insecurity during the MCO period. Fifth, acquiescence and recall biases are also common in a face-to-face survey, particularly when it is conducted among individuals with low socioeconomic backgrounds [77]. Moreover, a single dietary record may not actually reflect the children’s usual food intake pattern. Sixth, DDS was treated as an exogenous (independent) variable in the analyses. Even though we had performed the subsidiary analyses (Supplementary Table S2), the reverse causality between DDS and child hunger could not be sufficiently evaluated. To verify this, we suggest instrumental variables and two-stage least squares regression analysis (2SLS) techniques be employed in the future using a larger sample size or using panel data from a cohort study. In this way, the causal pathway between DDS and child hunger can be more appropriately elucidated by controlling the effects of other unmeasured confounders and reverse causation, which ultimately resolves the endogeneity issue between DDS and the error term in the regression equation. In order to achieve this, however, a large sample size or panel data from a cohort study are required to ensure unbiased and valid causality assessment. Seventh, the results could be made more accurate if the Minimum Dietary Diversity for Women (MDD-W) score [78], a new dietary diversity scoring system released by the FAO in 2021, was used to calculate the DDS for our study participants instead of the WDD scale. However, this was infeasible since we had concluded our research in January 2021, several months before the publication of the MDD-W by the FAO. Nevertheless, we aim to use the new MDD-W scale in our future research.

Finally, the predictive regression model should also be validated using an external sample or other statistical means, such as k-fold cross-validation or leave-one-out cross-validations procedures [79]. Obtaining an appropriate external sample for external validation might be a challenge due to the end of the COVID-19 lockdown in all countries globally, but this could be circumvented by using datasets that study investigators had collected in similar research settings and populations of interest.

## 5. Conclusions

The prevalence of household food insecurity, in particular child hunger, in an urban poor community in the Petaling administrative district reached an alarming level during the COVID-19 pandemic lockdown. Within households with food insecurity, we demonstrated a significant difference in the DDS between households experiencing child hunger and those without child hunger. Additionally, DDS is also a significant determinant of child hunger. Our study hence provides an insight into the consequences of food insecurity on these households and the children with respect to their overall development, food diversity, and nutritional status. However, further research is required to address our study limitations and thus verify our findings.

## Figures and Tables

**Table 1:** The calculated sample sizes for each determinant of child hunger.

Determinants	P_1_	P_0_^a^	*n*_group_ ^b^	*n*_final_ ^c^	*n*_total_
Mother’s education (primary education or less)	0.332	0.05	36	40	80
Father’s education (primary education or less)	0.511	0.200	42	47	94
Financial aid status	0.689	0.200	19	21	42

^a^ Based on the assumed minimum detectable effects (MDEs) in our setting; ^b^ Based on Fisher’s exact test; ^c^ Sample size (per group) after taking 10% attrition rate into consideration.

**Table 2:** The clinicodemographic background of households stratified by food insecurity status (*n* = 106).

Factors	Household and Individual Insecure (*n* = 44)	Child Hunger (*n* = 62)	χ^2^ Statistics (df) ^e^	*p*-Value
	Mean (SD)/*n* (%)	Mean (SD)/*n* (%)		
Child’s age at enrolment (months)	30.0 (20.5) ^a^	26.5 (27.3) ^a^	1158.5 ^b^	0.189 ^c^
Child’s gender			0.311 (1)	0.694
Male	21 (47.7)	33 (53.2)		
Female	23 (52.3)	29 (46.8)		
Preterm (<37 weeks)	8 (18.2)	8 (12.9)	0.560 (1)	0.583
SGA ^d^	5 (11.4)	11 (17.7)	0.817 (1)	0.421
Paternal age (years)	37.8 (7.58)	36.3 (7.44)	0.979 (100) ^f^	0.330
Maternal age (years)	35.0 (6.91)	33.3 (5.49)	1.370 (102) ^f^	0.174
Child’s ethnicity			0.799 (1)	0.485
Malay	39 (88.6)	58 (93.5)		
Non-Malay	5 (11.4)	4 (6.5)		
Paternal education			1.292 (2)	0.578
Post-Secondary	5 (11.6)	4 (6.7)		
Secondary	32 (74.4)	50 (83.3)		
Primary	6 (14.0)	6 (10.0)		
Maternal education			0.876 (3)	0.965
Post-Secondary	5 (11.4)	7 (11.3)		
Secondary	35 (79.5)	47 (75.8)		
Primary	4 (9.1)	7 (11.3)		
No formal education	0 (0.0)	1 (1.6)		
Paternal employment status			0.243 (1)	0.717
Employed	39 (90.7)	56 (93.3)		
Unemployed	4 (9.3)	4 (6.7)		
Maternal employment status			0.178 (1)	0.809
Employed	10 (22.7)	12 (19.4)		
Unemployed	34 (77.3)	50 (80.6)		
Number of employed parents			3.646 (2)	0.163
Both employed	5 (11.4)	10 (16.1)		
1 parent	39 (88.6)	48 (77.4)		
Both unemployed	0 (0.0)	4 (6.5)		
Total household income			1.570 (1)	0.272
≥RM 3000	5 (11.4)	3 (4.8)		
<RM 3000	39 (88.6)	59 (95.2)		
Financial aid recipient	21 (47.7)	37 (59.7)	1.483 (1)	0.241
Food aid recipient	34 (77.3)	50 (80.6)	0.178 (1)	0.809
Total number of children	2 (2) ^a^	3 (2) ^a^	1336.0 ^b^	0.857 ^c^

^a^ Median (IQR); ^b^ Mann–Whitney U statistics; ^c^ Exact Mann–Whitney test; ^d^ History of small for gestational age (SGA); ^e^ Degree of freedom; ^f^ t-statistic (Degree of freedom).

**Table 3:** The differences in the proportions of breastfeeding and SSB consumptions between toddlers and pre-school children aged 2 and above (*n* = 106).

Factors	Pre-School Children (*n* = 66)	Toddlers (*n* = 40)	χ^2^ Statistics (df)	*p*-Value
	*n* (%)	*n* (%)		
Breastfeeding	16 (24.2)	27 (67.5)	19.330 (1)	**<0.001**
Sugar-sweetened beverages	48 (72.7)	14 (46.8)	14.601 (1)	**<0.001**

**Table 4:** The differences in the children’s and parental anthropometric measurements between the combined household and individual insecure and child hunger groups (*n* = 106).

Factors	Household and Individual Insecure (*n* = 44)	Child Hunger (*n* = 62)	Mean Diff (95% CI)	χ^2^ (df)	*p*-Value
	Mean (SE)/*n* (%)	Mean (SE)/*n* (%)			
Height-for-age z-score ^a^	−1.152 (0.157) ^c^	−0.961 (0.129) ^c^	0.191 (−0.206, 0.589)	-	0.342
Weight-for-age z-score ^b^	−1.120 (0.206) ^d^	−1.046 (0.168) ^d^	0.074 (−0.446, 0.595)	-	0.777
Weight-for-height z-score ^a^	−0.682 (0.267) ^c^	−0.846 (0.216) ^c^	−0.164 (−0.837, 0.510)	-	0.631
Stunting ^e^	9 (20.5)	8 (12.9)		1.090 (1)	0.421
Underweight ^e^	12 (27.3)	18 (29.0)		0.039 (1)	>0.999
Wasting ^e^	9 (20.5)	15 (24.2)		0.205 (1)	0.814
Father’s BMI				3.103 (3)	0.395
Underweight	0 (0.0)	2 (3.5)			
Normal	10 (23.3)	19 (33.3)			
Overweight	13 (30.2)	13 (22.8)			
Obese	20 (46.5)	23 (40.4)			
Mother’s BMI				6.106 (3)	0.094
Underweight	0 (0.0)	2 (3.3)			
Normal	12 (27.9)	7 (11.7)			
Overweight	11 (25.6)	14 (23.3)			
Obese	20 (46.5)	37 (61.7)			

No significant statistical interactions were found between confounders and food insecurity groups for both ANOVA and ANCOVA models. All assumptions for ANOVA (linearity, homoscedasticity, and independence of the residuals) and ANCOVA (ANOVA assumptions + homogeneity of regression slope (smallest p_interacions_ = 0.096 for food insecurity ∗ birthweight z-score interaction)) were met. One outlier was found (Figure S1 Supplementary Materials) but was not removed since it is a true observation. ^a^ Adjusted for breastfeeding status and SSB consumption; ^b^ Adjusted for birthweight (covariate), breastfeeding status, and sugar sweetened beverage consumption; ^c^ Adjusted marginal means (standard error) and 95% CI based on three-way ANOVA; ^d^ Adjusted marginal means (standard error) and 95% CI based on ANCOVA (birthweight z-score (covariate) fixed at −0.619); ^e^ Based on univariable chi-square tests.

**Table 5:** The associations between different (**a**) WHO-IYCF (in children aged <24 months, *n* = 40) or (**b**) WDD (in pre-school children aged ≥24 months, *n* = 66) food groups and child hunger.

(a)				
Factors	Household and Individual Insecure	Child Hunger	χ^2^ Statistics (df)	*p*-Value
	Median (IQR)/*n* (%)	Median (IQR)/*n* (%)		
Toddlers (*n* = 40)	***n* = 13**	***n* = 27**		
Dietary diversity score	6 (2)	5 (1)	475.50 ^a^	**0.023**
Minimum dietary diversity ≥ 5	11 (84.6)	14 (51.9)	4.019 (1)	0.080
Breast milk	10 (76.9)	17 (63.0)	0.780 (1)	0.484
Grains & starchy foods	12 (92.3)	27 (100.0)	2.130 (1)	0.325
Beans & peas	4 (30.8)	2 (7.4)	3.756 (1)	0.075
Dairy products	6 (46.2)	18 (66.7)	1.538 (1)	0.305
Flesh foods	12 (92.3)	20 (74.1)	1.823 (1)	0.236
Eggs	8 (61.5)	14 (51.9)	0.333 (1)	0.737
Vitamin A rich fruits and vegetables	9 (69.2)	15 (55.6)	0.684 (1)	0.503
Other fruits and vegetable types	12 (92.3)	20 (74.1)	1.823 (1)	0.236
Sugar-sweetened beverages	5 (38.5)	9 (33.3)	0.101 (1)	>0.999
(**b**)				
**Factors**	**Household and Individual Insecure**	**Child Hunger**	**χ^2^ Statistics (df)**	***p*-Value**
	**Median (IQR)/*n* (%)**	**Median (IQR)/*n* (%)**		
Pre-school children (*n* = 66)	***n* = 31**	***n* = 35**		
Dietary diversity score	6 (1)	5 (2)	463.0 ^a^	0.294
Minimum dietary diversity ≥ 5	24 (77.4)	26 (74.3)	0.088 (1)	0.783
Starchy Staples	27 (87.1)	30 (85.7)	0.027 (1)	>0.999
Legumes and Nuts	11 (35.5)	7 (20.0)	1.987 (1)	0.178
Dairy products	27 (87.1)	29 (82.9)	0.230 (1)	0.739
Organ meats	2 (6.5)	3 (8.6)	0.106 (1)	>0.999
Eggs	24 (77.4)	25 (71.4)	0.309 (1)	0.779
Flesh food	24 (77.4)	29 (82.9)	0.307 (1)	0.758
Dark-green leafy vegetables	15 (48.4)	15 (42.9)	0.203 (1)	0.805
Vitamin A-rich vegetables and fruits	21 (67.7)	18 (51.4)	1.810 (1)	0.215
Other fruits and vegetables	17 (54.8)	23 (65.7)	0.814 (1)	0.452
Sugar-sweetened beverages	23 (74.2)	25 (71.4)	0.063 (1)	>0.999

^a^ Mann–Whitney u statistics.

**Table 6:** The determinants of child hunger among the households living in the People Housing Project (*n* = 103).

Determinants	Simple Logistic Regression				Multiple Logistic Regression			
	β (SE)	Crude Odds Ratio (95% CI)	Wald Statistics (df)	*p*-Value	β (SE)	Adjusted Odds Ratio (95% CI) ^c^	Wald Statistics (df)	*p*-Value
**Father’s highest education**			1.276 (2) ^b^	0.528			1.025 (2) ^b^	0.599
Post-Secondary	-	1 ^a^	-	-	-	1 ^a^	-	-
Secondary	0.669 (0.708)	1.953 (0.488, 7.823)	0.894 (1)	0.344	0.634 (0.720)	1.885 (0.459, 7.732)	0.775(1)	0.379
Primary	0.223 (0.885)	1.250 (0.221, 7.084)	0.064 (1)	0.801	0.235 (0.910)	1.265 (0.212, 7.530)	0.067 (1)	0.796
**Mother’s highest education**			0.375 (3) ^d^	0.945 ^d^			0.374 (3) ^d^	0.946 ^d^
Post-Secondary	-	1 ^a^	-	-	-	1 ^a^	-	-
Secondary	−0.019 (0.604)	0.981 (0.301, 3.203)	−0.030 (1)	0.975	0.169 (0.627)	1.185 (0.346,4.051)	0.27 (1)	0.787
Primary	0.201 (0.819)	1.222 (0.245, 6.085)	0.250 (1)	0.806	0.226 (0.853)	1.253 (0.236, 6.664)	0.26 (1)	0.769
No formal education	0.788 (1.727)	2.200 (0.075, 64.904)	0.460 (1)	0.633	0.823 (1.735)	2.276 (0.076, 68.219)	0.47 (1)	0.635
**Mother’s employment status**			0.178 (1)	0.673			0.143 (1)	0.706
Working	-	1 ^a^			-	1 ^a^		
Not Working	0.203 (0.482)	1.225 (0.476, 3.155)			0.206 (0.544)	1.228 (0.423, 3.570)		
**Total household income**			1.455 (1)	0.228			1.690 (1)	0.194
≥RM 3000	-	1 ^a^			-	1 ^a^		
<RM 3000	0.916 (0.760)	2.500 (0.564, 11.082)			1.003 (0.771)	2.725 (0.601, 12.359)		
**Financial aid**			1.475 (1)	0.224			1.748 (1)	0.186
Yes	-	1 ^a^			-	1 ^a^		
No	−0.483 (0.398)	0.617 (0.283, 1.345)			−0.548 (0.415)	0.578 (0.257, 1.303)		
**Dietary diversity score**	−0.369 (0.171)	0.691 (0.495, 0.966)	4.679 (1)	**0.031**	−0.451 (0.181)	0.637 (0.443, 0.916)	5.914 (1)	**0.015**
**Sugar-sweetened beverage**			0.818 (1)	0.366			0.430 (1)	0.512
No	-	1 ^a^			-	1 ^a^		
Yes	−0.365 (0.404)	0.694 (0.314, 1.532)			−0.277 (0.422)	0.758 (0.332. 1.734)		
**Mother’s age (years) ^e^**	−0.045 (0.033)	0.956 (0.895, 1.020)	1.851 (1)	0.174				
**Father’s Employment Status ^e^**			0.241 (1)	0.624				
Yes	-	1 ^a^						
No	0.362 (0.737)	1.436 (0.339, 6.090)						
**No of children (count) ^e^**	−0.004 (0.120)	0.996 (0.788, 1.260)	0.001 (1)	0.975				

Multiple Imputation was not carried out since the percentage of missing cases is 2.83% (threshold: 5%). Model building is based on forward and backward stepwise regression and purposeful selection of covariate methods. No multicollinearity (largest VIF = 1.146, condition index—18.969, tolerance = 0.873) and statistical interaction (smallest pinteraction = 0.067 for maternal age-number of children interaction) were detected. Satisfactory model fit (Hosmer–Lemeshow statistic (df): 10.582 (8), *p* = 0.227; 0.700 (95% CI: 0.590, 0.798, *p* = 0.001)). There were no influential observations (largest leverage = 0.1964 (threshold = 0.0377); however, no large-leverage observation has dfbeta more than 1). ^a^ Baseline group; ^b^ Likelihood ratio statistics (df); ^c^ Adjusted for maternal age, paternal employment status, the number of children in the household; ^d^ Based on changes in Firth’s penalized log-likelihood; ^e^ Theoretically important confounders whose effects imperatively need to be adjusted for.

## Data Availability

The data from this study are available on request from the corresponding author. The data are not publicly available due to patient confidentiality and are used under license for the current study.

## Supplementary Materials

The following supporting information could be downloaded at https://www.mdpi.com/article/10.3390/nu15102356/s1, Figure S1: Plots of residuals versus predicted values for (a) weight-for-age, (b) height-for-age and (c) weight-for-height z-scores, and (d) Q-Q plot for normality check; Table S1: Type of COVID-19 food relief received by the households; Table S2: The assessment of potential associations between child hunger’s potential determinants and DDS treated as an endogenous variable (*n* = 103). File S1: Patient information sheet and consent form (English version); File S2: Patient information sheet and consent form (Malay version); File S3: Demographic data collection sheet (English version); File S4: Demographic data collection sheet (Malay version); File S5: The validated Radimer-Cornell Food Security Questionnaire (Malay version); File S6: 24-h dietary recall log (English version); File S7: 24-h dietary recall log (Malay version).

## References

[1] Anderson S.A. (1990). Core Indicators of Nutritional State for Difficult-to-Sample Populations. J. Nutr..

[2] FAO (2003). Trade Reforms and Food Security: Conceptualizing the Linkages.

[3] Bickle G., Nord M., Price C., Hamilton W., Cook J. (2000). Guide to Measuring Household Food Security (Revised 2000).

[4] Shankar P., Chung R., Frank D.A. (2017). Association of Food Insecurity with Children’s Behavioral, Emotional, and Academic Outcomes: A Systematic Review. J. Dev. Behav. Pediatr..

[5] Barker D.J.P. (2007). The origins of the developmental origins theory. J. Intern. Med..

[6] Campisano S., La Colla A., Echarte S.M., Chisari A.N. (2019). Interplay between early-life malnutrition, epigenetic modulation of the immune function and liver diseases. Nutr. Res. Rev..

[7] Peter C., Fischer L.K., Kundakovic M., Garg P., Jakovcevski M., Dincer A., Amaral A.C., Ginns E.I., Galdzicka M., Bryce C.P. (2016). DNA Methylation Signatures of Early Childhood Malnutrition Associated With Impairments in Attention and Cognition. Biol. Psychiatry.

[8] Simonovich S.D., Pineros-Leano M., Ali A., Awosika O., Herman A., Withington M.H.C., Loiacono B., Cory M., Estrada M., Soto D. (2020). A systematic review examining the relationship between food insecurity and early childhood physiological health outcomes. Transl. Behav. Med..

[9] Ihab A., Rohana A., Manan W.W., Suriati W.W., Zalilah M., Rusli A. (2012). Food Expenditure and Diet Diversity Score are Predictors of Household Food Insecurity among Low Income Households in Rural District of Kelantan Malaysia. Pak. J. Nutr..

[10] Ruszczyk H.A., Rahman M.F., Bracken L.J., Sudha S. (2020). Contextualizing the COVID-19 pandemic’s impact on food security in two small cities in Bangladesh. Environ. Urban..

[11] Sulaiman N., Yeatman H., Russell J., Law L.S. (2021). A Food Insecurity Systematic Review: Experience from Malaysia. Nutrients.

[12] Choong C., Tan Z.G. (2019). The Absolute vs. Relative Poverty Conundrum.

[13] Khalid M.A., Rosli Z., Fatimahtul S.N., Halim M.A., Akbar E.S. (2018). Children without: A Study of Urban Child Poverty and Deprivation in Low-Cost Flats in Kuala Lumpur.

[14] National Institutes of Health, Ministy of Health Malaysia (2020). National Health and Morbidity Survey (NHMS) 2019: Non-Communicable Diseases, Healthcare Demand, and Health Literacy—Key Findings.

[15] Wang C.C., Jalal M.I.A., Song Z.L., Teo Y.P., Tan C.A., Heng K.V., Low M.S.Y., Zaini A.A., Lum L.C.S. (2022). A Randomized Pilot Trial of Micronutrient Supplementation for under-5 Children in an Urban Low-Cost Flat Community in Malaysia: A Framework for Community-Based Research Integration. Int. J. Environ. Res. Public Health.

[16] The Economist Intelligence Unit (2019). Global Food Security Index 2019 Strengthening Food Systems and the Environment through Innovation and Investment.

[17] The Economist Intelligence Unit (2020). Global Food Security Index 2020 Addressing Structure Inequalities to Build Strong and Sustainable Food Systems.

[18] Rahman T., Hasnain M.D.G., Islam A. (2021). Food insecurity and mental health of women during COVID-19: Evidence from a developing country. PLoS ONE.

[19] Department of Statistics, Malaysia (2020). Report of Special Survey on Effects of COVID-19 on Economy and Individual (Round 1). Kuala Lumpur, Malaysia.

[20] Ibrahim A.Z., Othman Z. (2020). COVID-19: Coping strategies among B40 households in Malaysia to achieve food security during movement control order (MCO). Eur. J. Mol. Clin. Med..

[21] Rivan N.F.M., Yahya H., Shahar S., Singh D.A., Ibrahim N., Ludin A.M., Sakian N.M., Mahadzir H., Subramaniam P., Kamaruddin M. (2021). The Impact of Poor Nutrient Intakes and Food Insecurity on the Psychological Distress among Community-Dwelling Middle-Aged and Older Adults during the COVID-19 Pandemic. Nutrients.

[22] Abdullah R.G., Mersat N.I., Wong S.-K. (2021). Implications of Covid-19 Pandemic on Household Food Security: Experience from Sarawak, Malaysia. Int. J. Bus. Soc..

[23] Ahmad A., Shahril M.R., Wan-Arfah N., Abu Bakar W.A.M., Piernas C., Lua P.L. (2022). Changes in health-related lifestyles and food insecurity and its association with quality of life during the COVID-19 lockdown in Malaysia. BMC Public Health.

[24] Department of Statistics Malaysia (2021). My Local Stats Malaysia 2021.

[25] Department of Statistics Malaysia (2021). My Local Stats Selangor 2021.

[26] Vandenbroucke J.P., von Elm E., Altman D.G., Gøtzsche P.C., Mulrow C.D., Pocock S.J., Poole C., Schlesselman J.J., Egger M., STROBE Initiative (2014). Strengthening the Reporting of Observational Studies in Epidemiology (STROBE): Explanation and elaboration. Int. J. Surg..

[27] Sharma A., Duc N.T.M., Thang T.L.L., Nam N.H., Ng S.J., Abbas K.S., Huy N.T., Marušić A., Paul C.L., Kwok J. (2021). A Consensus-Based Checklist for Reporting of Survey Studies (CROSS). J. Gen. Intern. Med..

[28] Tan S.T., Tan C.X., Tan S.S. (2022). Food Security during the COVID-19 Home Confinement: A Cross-Sectional Study Focusing on Adults in Malaysia. Hum. Nutr. Metab..

[29] Azizam N.S.N., Yusof S.N., Amon J.J., Ahmad A., Safii N.S., Jamil N.A. (2022). Sports Nutrition and Food Knowledge among Malaysian University Athletes. Nutrients.

[30] Calder B.J., Phillips L.W., Tybout A.M. (1981). Designing Research for Application. J. Consum. Res..

[31] Faugier J., Sargeant M. (1997). Sampling hard to reach populations. J. Adv. Nurs..

[32] Berndt A.E. (2020). Sampling Methods. J. Hum. Lact..

[33] Association W.M. (2013). World Medical Association Declaration of Helsinki: Ethical Principles for Medical Research Involving Human Subjects. JAMA.

[34] Naing L., Winn T., Rusli B.N. (2006). Practical Issues in Calculating the Sample Size for Prevalence Studies. Arch. Orofac. Sci..

[35] Dupont W.D., Plummer W.D. (1990). Power and sample size calculations: A review and computer program. Control. Clin. Trials.

[36] McIntyre L., Connor S.K., Warren J. (2000). Child hunger in Canada: Results of the 1994 National Longitudinal Survey of Children and Youth. Can. Med. Assoc. J..

[37] WHO Multicentre Growth Reference Standard Group (2006). WHO Child Growth Standards based on length/height, weight and age. Acta Paediatr..

[38] United Nations Children’s Fund (UNICEF), World Health Organization (WHO), Food And Nutrition Technical Assistance (FANTA III), United States Agency for International Development (USAID) (2017). Meeting Report on Considering, Refining and Extending the World Health Organization Infant and Young Child Feeding Indicators.

[39] Kennedy G., Ballard T., Dop M. (2010). Guidelines for Measuring Household and Individual Dietary Diversity.

[40] Martn-Prével Y., Allemand P., Wiesmann D., Arimond M., Ballard T., Deitchler M., Dop M.C., Kennedy G., Lee W.T., Mousi M. (2015). Moving forward on Choosing a Standard Operational Indicator of Women’s Dietary Diversity.

[41] World Health Organization (WHO), United Nations Children’s Fund (UNICEF) (2021). Indicators for Assessing Infant and Young Child Feeding Practices: Definitions and Measurement Methods.

[42] World Health Organization (WHO) (2017). Taxes on Sugary Drinks: Why Do It?.

[43] Sousa A., Sych J., Rohrmann S., Faeh D. (2020). The Importance of Sweet Beverage Definitions When Targeting Health Policies—The Case of Switzerland. Nutrients.

[44] Sharif Z.M., Ang M. (2001). Assessment of food insecurity among low income households in Kuala Lumpur using the Radimer/Cornell food insecurity instrument—A validation study. Malays. J. Nutr..

[45] Sharif Z.M. (1998). Growth Status Determinants of School Age Children from Primarily Low-Income Households in the Urban Area of Kuala Lumpur, Malaysia: A Focus on Intrahousehold Factors. Ph.D. Thesis.

[46] Radimer K.L., Olson C.M., Campbell C.C. (1990). Development of Indicators to Assess Hunger. J. Nutr..

[47] Radimer K.L., Olson C.M., Greene J.C., Campbell C.C., Habicht J.-P. (1992). Understanding hunger and developing indicators to assess it in women and children. J. Nutr. Educ..

[48] van Buuren S., Groothuis-Oudshoorn K. (2011). Mice: Multivariate Imputation by Chained Equations in R. J. Stat. Soft..

[49] Jakobsen J.C., Gluud C., Wetterslev J., Winkel P., Jakobsen J.C., Gluud C., Wetterslev J., Winkel P. (2017). When and how should multiple imputation be used for handling missing data in randomised clinical trials—A practical guide with flowcharts. BMC Med. Res. Methodol..

[50] Mehta C.R., Patel N.R. (2013). IBM SPSS Exact Test.

[51] Hosmer J.R.D.W., Lemeshow S., Sturdivant R.X. (2013). Applied Logistic Regression.

[52] Firth D. (1993). Bias reduction of maximum likelihood estimates. Biometrika.

[53] Jeyaseelan L., Devika S., Sebastian G. (2016). Analysis of sparse data in logistic regression in medical research: A newer approach. J. Postgrad. Med..

[54] Naser I.A., Jalil R., Muda W.M.W., Nik W.S.W., Shariff Z.M., Abdullah M.R. (2014). Association between household food insecurity and nutritional outcomes among children in Northeastern of Peninsular Malaysia. Nutr. Res. Pract..

[55] Sharkey J.R., Dean W.R., Nalty C.C. (2013). Child hunger and the protective effects of supplemental nutrition assistance program (SNAP) and alternative food sources among Mexican-origin families in Texas border colonias. BMC Pediatr..

[56] Stoltzfus J.C. (2011). Logistic Regression: A Brief Primer. Acad. Emerg. Med..

[57] Tabachnick B.G., Fidell L.S. (2019). Using Multivariate Statistics.

[58] Hair J.F., Black W.C., Babin B.J., Anderson R.E. (2019). Multivariate Data Analysis.

[59] Martín N., Pardo L. (2009). On the asymptotic distribution of Cook’s distance in logistic regression models. J. Appl. Stat..

[60] FAO, IFAD, UNICEF, WFP, WHO (2021). The State of Food Security and Nutrition in the World 2021. Transforming Food Systems for Food Security, Improved Nutrition and Affordable Healthy Diets for All.

[61] Rengarajoo S., Tan S.T. (2022). Household Income and its Correlation with Child Hunger during the COVID-19 Pandemic: A Cross-Sectional Study. J. Hunger Environ. Nutr..

[62] Singh S., Sagar R. (2021). A critical look at online survey or questionnaire-based research studies during COVID-19. Asian J. Psychiatry.

[63] Heerwegh D. (2009). Mode Differences between Face-to-Face and Web Surveys: An Experimental Investigation of Data Quality and Social Desirability Effects. Int. J. Public Opin. Res..

[64] Heerwegh D., Loosveldt G. (2008). Face-to-Face versus Web Surveying in a High-Internet-Coverage Population: Differences in Response Quality. Public Opin. Q..

[65] Szolnoki G., Hoffmann D. (2013). Online, face-to-face and telephone surveys—Comparing different sampling methods in wine consumer research. Wine Econ. Policy.

[66] El Bilbeisi A.H., Al-Jawaldeh A., AlBelbeisi A., Abuzerr S., Elmadfa I., Nasreddine L. (2022). Households’ Food Insecurity and Its Association with Demographic and Socioeconomic Factors in Gaza Strip, Palestine: A Cross-Sectional Study. Ethiop. J. Health Sci..

[67] Tariqujjaman, Rahman M., Wangdi K., Karmakar G., Ahmed T., Sarma H. (2023). Geographical variations of food insecurity and its associated factors in Bangladesh: Evidence from pooled data of seven cross-sectional surveys. PLoS ONE.

[68] Murdad R., Muhiddin M., Osman W.H., Tajidin N.E., Haida Z., Awang A., Jalloh M.B. (2022). Ensuring Urban Food Security in Malaysia during the COVID-19 Pandemic—Is Urban Farming the Answer? A Review. Sustainability.

[69] Cho Y., Johnson D., Kawasoe Y., Avalos J., Rodriguez R. The Impact of the COVID-19 Crisis on Low Income Households in the Philippines: Deepening Distress Despite Rebounding Economy. COVID-19 Low Income HOPE Survey Note, (2). The World Bank. https://documents.worldbank.org/curated/en/768871611898266913/pdf/The-Impact-of-the-COVID-19-Crisis-on-Low-Income-Households-in-the-Philippines-Deepening-Distress-Despite-Rebounding-Economy.pdf.

[70] Nepali S., Simkhada P., Davies I.G. (2020). Association between wasting and food insecurity among children under five years: Findings from Nepal demographic health survey 2016. BMC Public Health.

[71] Pathak J., Mahanta T.G., Arora P., Kalita D., Kaur G. (2020). Malnutrition and household food insecurity in children attending anganwadi centres in a district of North East India. Indian J. Community Med..

[72] Ali N.B., Tahsina T., Hoque D.M.E., Hasan M.M., Iqbal A., Huda T.M., El Arifeen S. (2019). Association of food security and other socio-economic factors with dietary diversity and nutritional statuses of children aged 6-59 months in rural Bangladesh. PLoS ONE.

[73] Sambu W.C., Hall K., Roelen K., Morgan R., Tafere Y. (2019). Chapter 3: Poverty and Child Hunger in South Africa: A Child-Centred Analysis of Household-Level Survey Data. Putting Children First: New Frontiers in the Fight against Child Poverty in Africa.

[74] Porter C., Goyal R. (2016). Social protection for all ages? Impacts of Ethiopia’s Productive Safety Net Program on child nutrition. Soc. Sci. Med..

[75] Makate M., Nyamuranga C. (2023). The long-term impact of education on dietary diversity among women in Zimbabwe. Rev. Dev. Econ..

[76] Anane I., Nie F., Huang J. (2021). Socioeconomic and Geographic Pattern of Food Consumption and Dietary Diversity among Children Aged 6–23 Months Old in Ghana. Nutrients.

[77] Benson J., Garrison E., Dropkin J., Jenkins P.L. (2016). Methodological concerns related to response bias in migrant and seasonal farmworkers. Am. J. Ind. Med..

[78] Food and Agriculture Organization (FAO) (2021). Minimum Dietary Diversity for Women an Updated Guide for Measurement: From Collection to Action.

[79] James G., Witten D., Hastie T., Tibshirani R. (2021). An Introduction to Statistical Learning: With Applications in R.

