# Supplementary material for: The Prevalence and Determinants of Child Hunger and Its Associations with Early Childhood Nutritional Status among Urban Poverty Households during COVID-19 Pandemic in Petaling District, Malaysia: An Exploratory Cross-Sectional Survey
Source: Nutrients. 2023 May 17;15(10):2356. doi: 10.3390/nu15102356 (PMC10222894; doi:10.3390/nu15102356)
Supplement: Supplementary file 1 [file nutrients-15-02356-s001.zip › nutrients-2315673-supplementary.pdf]

Table S1: Type of COVID-19 food relief received by the households.

Type of food aid received	Overall (n=84)	Household insecure (n=15)	Individual insecure (n=19)	Child hunger (n=50)
	n (%)	n (%)	n (%)	n (%)
Rice	80 (95.2)	13 (86.7)	18 (94.7)	49 (98.0)
Milk powder	63 (75.0)	14 (93.3)	16 (84.2)	33 (66.0)
Sugar	79 (94.0)	14 (93.3)	18 (94.7)	47 (94.0)
Cooking oil	80 (95.2)	14 (93.3)	18 (94.7)	48 (96.0)
Others	42 (50.0)	7 (46.7)	11 (57.9)	24 (48.0)

Table S2: The assessment of potential associations between child hunger's potential determinants and DDS treated as an endogenous variable (n=103).

Determinants	Simple linear regression				Multiple linear regression[d]			
	β (SE)	95% CI β	t-statistics	p-value	Adjusted β (SE)	95% CI Adjusted β	t-statistics	p-value
**Father's highest education**			0.809 (2,100)[b]	0.448[c]			0.831 (2,95)[e]	0.439[f]
Post-Secondary	-	-[a]			-	-[a]		
Secondary	-0.557 (0.445)	-1.439, 0.325	-1.253	0.213	-0.575 (0.454)	-1.476, 0.735	-1.266	0.209
Primary	0.417 (0.558)	-1.524, 0.691	-0.746	0.457	-0.406 (0.575)	-1.546, 0.375	-0.706	0.482
**Mother's highest education**			1.251 (3,99)[b]	0.295[c]			1.218 (3,94)[e]	0.307[f]
Post-Secondary	-	[a]			-	1[a]		
Secondary	-0.125 (0.405)	-0.927, 0.678	-0.308	0.759	-0.153 (0.416)	-0.979, 0.672	-0.369	0.713
Primary	0.227 (0.550)	-0.864, 1.319	0.413	0.680	0.248 (0.569)	-0.882, 1.378	0.436	0.664
No formal education	-2.273 (1.315)	-4.882, 0.336	-1.728	0.087	-2.227 (1.339)	-4.886, 0.432	-1.663	0.100
**Mother's employment status**							0.143 (1)	0.706
Working	-	-[a]			-	1[a]		
Not Working	-0.155 (0.316)	-0.782, 0.471	-0.492	0.624	-0.096 (0.346)	-0.782, 0.591	-0.276	0.783
**Total Household Income**								
**≥RM 3000**								
**< RM 3000**	-	-[a]			-	-[a]		
	0.189 (0.467)	-0.737, 1.116	0.406	0.686	0.134 (0.479)	-0.818, 1.085	0.279	0.781
**Financial aid**								
Yes	-	-[a]			-	-[a]		
No	0.234 (0.251)	-0.263, 0.731	0.934	0.352	0.200 (0.259)	-0.316, 0.775	0.769	0.444
**Sugar-sweetened beverage**								
No	-	-[a]			-	-[a]		

Yes	0.220 (0.253)	-0.281, 0.722	0.871	0.386	0.212 (0.263)	-0.310, 0.734	0.805	0.423
**Mother's age (years)[e]**	0.011 (0.021)	-0.030, 0.053	0.535	0.594				
**Father's Employment Status[e]**								
Yes	-	-[a]						
No	0.353 (0.466)	-0.572, 1.277	0.756	0.451				
**No of children (count)[e]**	-0.024 (0.077)	-0.177, 0.128	-0.316	0.753				

[a]Baseline reference group; [b]F statistics (between-group degree of freedom (k-1) where k = number of levels within the category, error degree of freedom (n-k-1). For categorical variables with more than 2 levels only; [c]Based on F-statistics, testing for the omnibus significance of the predictor. [d]Adjusted for maternal age, paternal employment status, the number of children in the household (confounders); [e]F statistics, adjusted for all confounders (between-group degree of freedom (k-1), error degree of freedom (n-$\sum individual\ df\ \ for\ all\ model\ preditors$). For categorical variables with more than 2 levels only;[f]Based on adjusted F statistics..

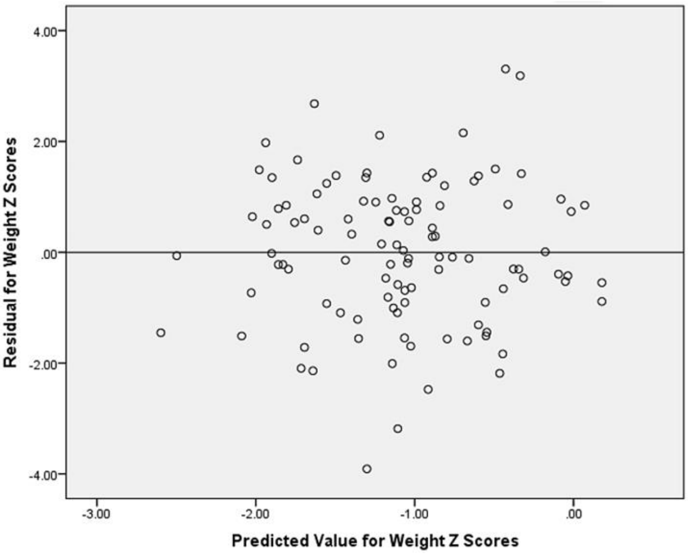

a) Weight-for-age z-scores

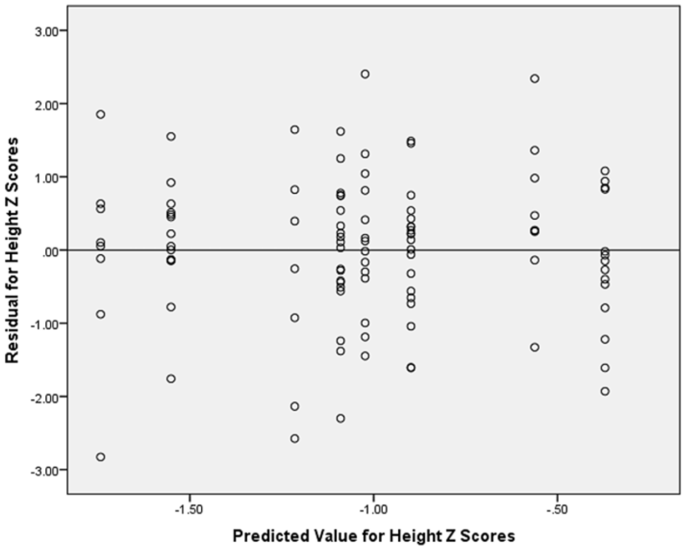

b) Height-for-age z-scores

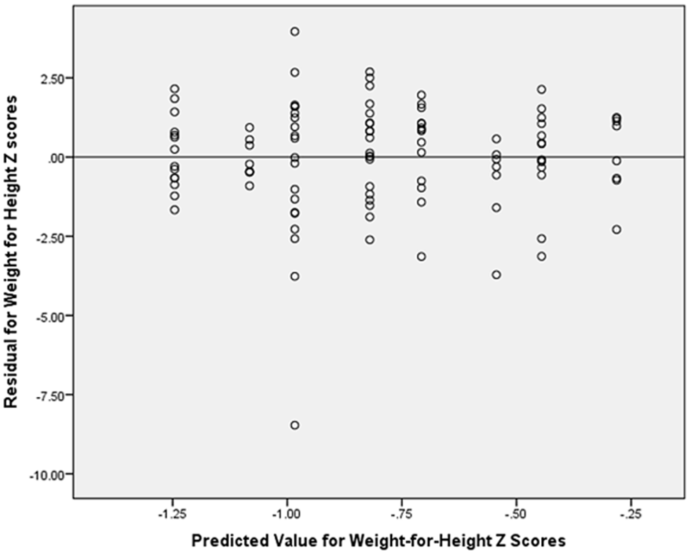

c) Weight-for-height z-scores

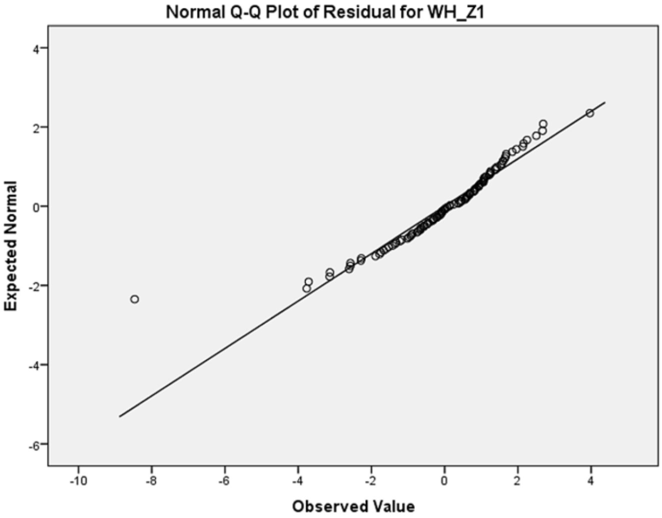

d) Q-Q plot for normality for weight-to-height z-scores

Figure S1: Plots of residuals versus predicted values for (a) weight-for-age, (b) height-for-age and (c) weight-for-height z-scores, and (d) Q-Q plot for normality check. Random scatter and no obvious pattern were found in each plot, demonstrating homoscedasticity, linearity and independence of residuals. One outlier was found in c) (bottom point) but the normality assumption was not severely affected as evidenced by the satisfactory Q-Q plot in d) and thus the outlier was retained in the model since it is a true observation.

Supplementary File S1: Patient information sheet and consent form (English version).

PATIENT INFORMATION AND INFORMED CONSENT

Please read the following information carefully, and do not hesitate to discuss any questions you may have with your doctor.

Study Title: Dietary Diversity and Iron Status of Young Children

Introduction:

An adequate variety of food is important to meet the macro- and micronutrient requirements for the growth and development of a child, especially in the first 1000 days of life. Suboptimal daily nutrient intake in this period may result in nutritional deficiencies and chronic malnutrition. Iron deficiency which can lead to anemia, is the commonest micronutrient deficiency. Young children are particularly vulnerable, and it is essential to monitor their dietary diversity.

What is the purpose of this study?

The purpose of the study is to evaluate the diversity of diet in young children and its effect on growth parameters as well as hemoglobin and iron status. The food security status of your family will be assessed together.

What are the procedures to be followed?

Your child is eligible for the study if he/she is aged between 6 months to 7 years old. You will be interviewed by a trained study coordinator to determine the dietary practices of your child and family. You will be asked to fill out a questionnaire on the food security status of your family. The height and weight of your child will be measured at baseline and subsequent follow-ups. In addition, a 2 ml blood sample for hemoglobin and iron status will be required at the start and 6 months later in the study if necessary. Follow-up telephone calls on dietary assessment will be done from time-to-time basis.

What will be the benefits of the study?

Detailed dietary intake, food security, and growth parameters will be assessed. You will benefit from nutritional advise given to your child which is essential for their optimal physical and mental development. Your child’s hemoglobin and iron status will also be known, and early treatment can be given if any deficiency is identified.

What are the possible drawbacks?

A detailed dietary questionnaire, food security status, along with weight and height measurement will be collected which can take up to 15 to 20 minutes of your time. We will require your commitment to participate in the follow-up telephone call on the dietary component by our trained community research assistants for the success of this study. Your child might experience some discomfort during the blood-taking procedure. We will give a spray of stop-pain medication before the blood test.

Can I refuse to take part in the study?

Yes. Your participation is completely voluntary, and we would encourage you to take part in this study. However, if you do not want to be in the study, your decision will not in any way interfere with your child’s baseline health.

Whom should I contact if I have additional questions during the course of the study?

Study Coordinator : Prof Dr Lucy Lum

Contact : 013-390 7898

Who should I contact if I have any problems and concerns for the study?

Medical Research Ethics Committee, University of Malaya Medical Centre

Telephone number: 03-7949 3209/2251

CONSENT BY PATIENT FOR CLINICAL RESEARCH

Nombor Versi: 2
Tarikh Versi: 10/04/2020

I, …………………………………………………………………, Identity Card No.: ……………………………………
(Name of Parent)

And my child:………………………………………………….., MyKid No: ………………………………………………..

of ………..
(Address)

hereby agree to take part in the clinical research (clinical study/questionnaire study) specified below:

<u>Title of Study:</u> Dietary Diversity and Iron Status of Young Children

the nature and purpose of which has been explained to me by Dr. …………………………………..………………
(Name & stamp; Designation of Doctor)

, and interpreted by ……………………..…………… to the best of his/her ability in …………… language/dialect.
(Name & stamp; Designation of Interpreter)

I have been told about the nature of the clinical research in terms of methodology, possible adverse effects, and complications (as per the patient information sheet). After knowing and understanding all the possible advantages and disadvantages of this clinical research, I voluntarily consent of my own free will to participate in the clinical research specified above.

I understand that I can withdraw from this clinical research at any time without assigning any reason whatsoever, and in such a situation shall not be denied the benefits of usual treatment by the attending doctors.

Date : ……………………..

Signature/ Thumbprint : ………………………………………
(Parent)

IN THE PRESENCE OF

Name : ………………………………
Identify Card No. : ……………………………………………….

Signature : ………………………………………
(Witness for Signature of Parent)

Designation : ………..………………………………….

I confirm that I have explained to the patient the nature and purpose of the above-mentioned clinical research.

Date : ……..………………..

Signature : ………………………………………
(*Attending Doctor)*

R.**N.**

CONSENT BY PATIENT **Name**

FOR **Sex**

CLINICAL RESEARCH **Age**

Unit

BK-MIS-1117-E02

Supplementary File S2: Patient information sheet and consent form (Malay version).

MAKLUMAT DAN PERSETUJUAN TERMAKLUM PESAKIT

Sila baca maklumat ini dengan teliti, jika terdapat sebarang kemusykilan atau pertanyaan, sila berhubung dan berbincang dengan doktor anda.

Tajuk Kajian: Kepelbagaian Makanan, dan Status Kandungan Zat Besi Kanak-kanak

Pengenalan:

Nutrisi seimbang adalah penting untuk membantu dalam perkembangan fizikal dan mental terutama di kalangan kanak-kanak kecil. Dalam 1000 hari pertama, adalah sangat perlu untuk memastikan kanak-kanak mendapat nutrisi yang cukup dan seimbang terutamanya zat besi.

Apakah tujuan kajian ini?

Tujuan kajian ini diadakan adalah untuk menentukan sama ada kepelbagaian makanan dibekalkan dengan seimbang kepada anak-anak. Kandungan zat besi dan diagnosis of anemia juga akan dikaji bersamaan dengan sekuriti makanan.

Apakah prosedur yang perlu diikuti?

Anak anda layak menyertai kajian ini jika dia berumur antara 6 bulan hingga 7 tahun. Anda akan ditemu bual oleh pembantu kajian untuk menentukan amalan pemakanan (*diet*) anak dan keluarga anda. Anda juga diminta untuk mengisi soal selidik sekuriti makanan. Selepas itu, panggilan telefon akan dibuat dari masa ke semasa untuk mengetahui perkembangan makanan semasa. Ketinggian dan berat badan anak anda akan diukur. Disamping itu, satu sampel darah sebanyak 2ml (jumlah bersamaan dengan satu sudu kecil) akan diambil dari anak anda. Sampel darah yang kedua akan diambil sekiranya diperlukan atas perubahan dalam pemakanannya dan nasihat pakar perunding.

Apa manfaat yang akan diperolehi melalui kajian ini?

Amalan pemakanan anak anda dan sekuriti makanan isi rumah akan dikaji. Nasihat tentang pemakanan seimbang akan diberi. Tumbesarannya juga akan dipantau dengan teliti. Status kandungan zat besi dan hemoglobin anak anda akan diketahuai melalui keputusan darah dan dapat merawat dengan cepat dan lebih kesan.

Apakah kelemahan yang mungkin dihadapi?

Temubual mengenai amalan pemakanan, sekuriti makanan dan pengukuran ketinggian dan berat akan mengambil masa dalam maksimum 15-20 minit. Komitmen dari pihak keluarga untuk mendapat maklumbalas panggilan dari masa ke semasa amatlah diperlukan. Anak anda mungkin akan berasa kurang selesa semasa proses pengambilan darah. Kami akan membantu dengan memberikan semburan ubat tahan sakit sebelum pangamibilan darah.

Bolehkah saya tidak bersetuju untuk mengambil bahagian dalam kajian ini?

Ya. Penyertaan anda adalah secara sukarela dan kami menggalakkan anda untuk mengambil bahagian dalam kajian ini. Walau bagaimanapun, jika anda tidak bersetuju untuk mengambil bahagian dalam kajian ini, keputusan anda tidak akan mengganggu penjagaan yang akan anda/anak anda terima.

Siapakah yang perlu saya hubungi jika saya mempunyai persoalan sepanjang kajian ini?

Koordiantor kajian : Prof Dr Lucy Lum

Nombor telefon : 013-390 7898

Siapakah yang perlu saya hubungi jika ada masalah dengan kajian ini?

Medical Research Ethics Committee, University of Malaya Medical Centre

Nombor telefon: 03-7949 3209/2251

KEIZINAN OLEH PESAKIT UNTUK PENYELIDIKAN KLINIKAL

Nombor Versi: 2
Tarikh Versi: 10/04/2020

Saya, …………………..…………………………………………., No. Kad Pengenalan …………………..…………………
(Nama Ibu-bapa)

Dan anak saya……………………………..……………………., No. MyKid ……………………..………………………

beralamat…………………………………………………..………..…………………………………………..…………….
(Alamat)

dengan ini bersetuju menyertai dalam penyelidikan klinikal (pengajian klinikal/pengajian soal-selidik) disebut berikut:

Tajuk Penyelidikan: Kepelbagaian Makanan dan Status Kandungan Zat Besi Kanak-kanak

yang mana sifat dan tujuannya telah diterangkan kepada saya oleh Dr.…………………………………………..…………
(Nama & Jawatan Doktor)

mengikut terjemahan …………………..……………………………… yang telah menterjemahkan kepada saya dengan
(Nama & Jawatan Penterjemah)

sepenuh kemampuan dan kebolehannya di dalam Bahasa / loghat…………………………………………………………

Saya telah diberitahu bahawa dasar penyelidikan klinikal dalam keadaan methodologi, risiko dan komplikasi (mengikut kertas maklumat pesakit). Selepas mengetahui dan memahami semua kemungkinan kebaikan dan keburukan penyelidikan klinikal ini, saya merelakan/mengizinkan sendiri menyertai penyelidikan klinikal tersebut di atas.

Saya faham bahawa saya boleh menarik diri dari penyelidikan klinikal ini pada bila-bila masa tanpa memberi sebarang alasan dalam situasi ini dan tidak akan dikecualikan dari kemudahan rawatan dari doktor yang merawat.

Tarikh: ……………………..

Tandatangan/Cap Jari ……………………………………………….
(Ibu-bapa)

DI HADAPAN

Nama : ………………………………………………
No. K/P : …………………………………………….

Tandatangan : ……………………………………
(Saksi untuk Tandatangan Pesakit)

Jawatan : …………..…………………………….

Saya sahkan bahawa saya telah menerangkan kepada pesakit sifat dan tujuan penyelidikan klinikal tersebut di atas.

Tarikh: ……..…………………. Tandatangan ……………………………….
(Doktor yang merawat)

No. Pend.

KEIZINAN OLEH PESAKIT **Nama**
UNTUK **Jantina**
PENYELIDIKAN KLINIKAL **Umur** **Unit**

BK-MIS-1117-E02

Supplementary File S3: Demographic data collection sheet (English version).

SECTION A: DEMOGRAHIC DETIALS

DATA COLLECTION SHEET

Child's name : ……………………………………………………………………………………………..………..…

Fathers' name : ……………………………………………………..……………………………………..……

Fathers' age : ………………………………… Father's weight/ height: …………kg …………cm

Phone No. : ……………………………………………

Mothers' name : ……………………………………………………………………………………………………

Mothers' age : ………………………………… Mother's weight/ height: …………kg …………cm

Phone No. : ……………………………………………

Address : ……………………………………………………………………………………………………

FAMILY BACKGROUND

1) Race: Malay [ ] Chinese [ ] Indian [ ]
   Others [ ]; Please state: …………………………………………………….

2) Occupation:

Father	Unemployed	[ ]	Self-employed	[ ]	Employed	[ ]
Mother	Unemployed	[ ]	Self-employed	[ ]	Employed	[ ]

3) Education level:

Father	UPSR	[ ]	PMR	[ ]	SPM	[ ]
	Diploma	[ ]	Degree	[ ]	Post Grad	[ ]
	No formal education	[ ]				
Mother	UPSR	[ ]	PMR	[ ]	SPM	[ ]
	Diploma	[ ]	Degree	[ ]	Post Grad	[ ]
	No formal education	[ ]				

4) Total combined household income:

< RM 3,000	[ ]
RM 3,000 – RM 6,500	[ ]
RM 6,500 – RM 16,000	[ ]
> RM 16,000	[ ]

5) Family status:

Nucleus/ Extended family; Biological [ ] Adopted [ ]

Single-parent family [ ]

6) Total No. of children: (Age)

1

2

3

4

5

≥ 5 *(Please state accoridngly)*

Supplementary File S4: Demographic data collection sheet (Malay version).

SEKSYEN A: DEMOGRAFI

BORANG PENGUMPULAN DATA

Nama anak : ……………………………………………………………………………………………………..………

Nama bapa : ……………………………………………………..………………………………………………………

Umur bapa : ………………………………… Berat/tinggi bapa: …………kg …………cm

No. telefon : ……………………………………………

Nama ibu : ……

Umur ibu : ………………………………… Berat/tinggi ibu: …………kg …………cm

No. telefon : ……………………………………………

Alamat : ……

MAKLUMAT KELAURGA

1) Kaum: Melayu [ ] Cina [ ] India [ ]
Lain-lain [ ]; Sila nyatakan: ……………………………………………….

2) Pekerjaan:

Bapa	Tidak bekerja	[ ]	Bekerja sendiri	[ ]	Perkejaan bergaji	[ ]
Ibu	Tidak bekerja	[ ]	Bekerja sendiri	[ ]	Perkejaan bergaji	[ ]

3) Tahap pendidikan:

Bapa	UPSR	[ ]	PMR	[ ]	SPM	[ ]
	Diploma	[ ]	Degree	[ ]	Post Grad	[ ]
	Tiada pendidikan rasmi		[ ]			
Ibu	UPSR	[ ]	PMR	[ ]	SPM	[ ]
	Diploma	[ ]	Degree	[ ]	Post Grad	[ ]
	Tiada pendidikan rasmi		[ ]			

4) Jumlah pendapatan keseluruhan isi rumah:

< RM 3,000	[ ]
RM 3,000 – RM 6,500	[ ]
RM 6,500 – RM 16,000	[ ]
> RM 16,000	[ ]

5) Status keluarga:

Keluarga nucleus/ luas; Anak kandung [ ] Anak angkat [ ]

Keluarga induk tunggal [ ]

6) Jumlah anak: (Umur)

1

2

3

4

5

≥ 5 *(Sila nyatakan secara turutan)*

Supplementary File S5: The validated Radimer-Cornell Food Security Questionnaire (Malay version).

Soal Selidik Sekuriti Makanan Radimer-Cornell (Versi Bahasa Melayu)

Bagaimanakah keadaan makanan di dalam keluarga anda?

Bagi tiap-tiap soalan, bulatkan hanya satu sahaja nombor yang paling sesuai menerangkan keadaan tersebut. Berikut adalah keterangan mengenai nombor-nombor tersebut:

1: Tidak betul **atau** Tidak pernah terjadi
2: Kadangkala betul **atau** Kadang-kadang pernah terjadi
3: Selalu betul **atau** Selalu terjadi

Soalan

1. Saya risau sekiranya makanan atau bahan-bahan mentah untuk dimasak habis sebelum saya sempat mempunyai wang untuk membeli bekalan makanan lain.

1 2 3

2. Makanan atau bahan-bahan mentah untuk dimasak yang saya beli selalu cepat habis dan saya tidak mempunyai wang untuk membeli bekalan makanan lain.

1 2 3

3. Makanan atau bahan-bahan mentah untuk dimasak sebagai sajian keluarga (makan pagi, makan tengahari dan makan malam) tidak cukup dan saya tidak mempunyai wang untuk membeli bekalan makanan lain.

1 2 3

4. Kami sekeluarga makan makanan/lauk yang sama untuk beberapa hari berturut-turut kerana kami hanya mempunyai jenis makanan yang terhad (kurang pelbagai) dan kami tidak mempunyai wang untuk membeli bekalan makanan lain.

1 2 3

5. Saya tidak makan dengan kenyang / puas kerana tidak mampu untuk mendapatkan makanan.

1 2 3

6. Saya selalu berasa lapar tetapi saya tidak makan kerana tidak mampu untuk mendapatkan makanan.

1 2 3

7. Saya hanya makan sedikit sahaja daripada apa yang sepatutnya saya makan kerana saya tidak mampu untuk mendapatkan makanan.

1 2 3

8. Saya tidak dapat memberi sajian seimbang* kepada anak-anak saya kerana tidak mampu untuk mendapatkan makanan yang berzat.

1 2 3

(*seimbang – sajian yang mempunyai cukup (kuantiti dan kualiti) untuk bijirin (nasi), sayur, sumber haiwan atau protin)

9. Anak-anak saya tidak makan dengan cukup kerana saya tidak mampu untuk mendapatkan makanan yang cukup.

1 2 3

10. Saya tahu anak-anak saya berasa lapar tetapi saya tidak mampu untuk mendapatkan makanan yang cukup.

1 2 3

Supplementary File S6: 24-hour dietary recall log (English version).

24-HOUR DIETARY RECALL LOG

Child's name :

RN :

Date :

Researcher's name :

	Total food group :<br>Food allergy :	Morning	Mid-morning	Noon	Afternoon	Night	Yes	No	Not sure
1a	**(CARBOHYDRATE)** Porridge, rice, noodles, bread, spaghetti/pasta, thosai, roti canai						1	2	8
1b	**(CARBOHYDRATE)** White potatoes, white yam, tubers, tuberous roots						1	2	8
2	**(PROTEIN)** Beans, peas, lentils, nuts, seeds, dhal, tofu, tempeh						1	2	8
3	Breastmilk						1	2	8
4a	**(DAIRY PRODUCT)** Infant formula; Brand: ________<br><br>Total in 24-hours: ____ oz X _____ times						1	2	8
4b	**(DAIRY PRODUCT)** Cheese, yogurt, yogurt drinks						1	2	8

5a	**(PROTEIN)** Beef, pork, lamb, goat, chicken, duck						1	2	8
5b	**(PROTEIN)** Organ meats; liver, kidney, heart, or any others						1	2	8
5c	**(PROTEIN)** Fish, shellfish, prawns, crabs, or any other seafood						1	2	8
6	**(PROTEIN)** Egg						1	2	8
7a	**Vitamin A-rich vegetables:** Carrots, sweet potatoes, pumpkin						1	2	8
7b	**Vitamin A-rich fruits:** Ripe mangoes, ripe papayas, bananas, pineapples, oranges, nangka, chempedak						1	2	8
8a	**Dark green leafy vegetables:** Sawi, bayam, kailan, kangkong						1	2	8
8b	**Any other vegetables/ fruits:** cucumber, aubergines, apples						1	2	8
	Others								

		Morning	Mid-morning	Noon	Afternoon	Night	Yes	No	Not sure
9a	**Cooking oil:** Butter, olive oil, coconut milk						1	2	8
9b	**Processed food:** French fries, hotdogs, nuggets						1	2	8

10	**Sugary food:** Chocolates, sweets, cakes, sweet biscuits						1	2	8
11	**Sugary beverages**: cordial drinks, soft drinks, carbonated drinks, rose syrup water, chocolate malt *beverage*						1	2	8
12	**Packaged food**: biscuits, instant noodles, baby food						1	2	8
13	**Fresh fruit juice**						1	2	8
14	Condiments/ Herbs/ Spices/ Seasoning: ketchup, chili, ginger, soy sauce, fish sauce, peppers, salt, monosodium glutamate						1	2	8
16	How many times in a day that your child eat solid, semi-solid, or soft food other than liquid food?	Total times in day [ ]<br>Not sure: [ ]							
17	Does your child take any vitamin or mineral supplements?	Yes [ ]; Please state:________							
18	Does your child's food need to be grounded or blended (pureed)?	Yes [ ] No [ ]<br>Type of food: _______________							
19	Whether your child is spoon-feeding or self-feeding?	Spoon-feeding [ ] Self-feeding [ ] Mixed [ ]							

20	Whether your child eat together with the family?	Yes [ ] No [ ]

Duration of exclusive breastfeeding :

Main caregiver :

Total screen time in a day :

Storytelling activity : Yes [ ] No [ ]

Gestational age :

Birth weight : ………………..g; Length : ………………..cm; Head circumference : ………………..cm

Current weight : ………………..g; Current length/height : ………………..cm; Current head circumference : ………………..cm

Mother’s weight and height : ………………..kg; ………………..cm

Father’s weight and height : ………………..kg; ………………..cm

Supplementary File S7: 24-hour dietary recall log (Malay version).

MAKANAN YANG DIAMBIL DALAM MASA 24 JAM

Nama kanak-kanak :

RN :

Tarikh :

Nama penyelidik :

	Jumlah kumpulan makanan : Alahan makanan :	Pagi	Tengah pagi	Tengahari	Petang	Malam	Ya	Tidak	Tidak tahu
1a	**(KARBOHIDRAT)** Bubur, nasi, mi, roti, spaghetti/pasta, thosai, roti canai						1	2	8
1b	**(KARBOHIDRAT)** Kentang putih, keladi putih, atau makanan lain yang sumbernya dari akar						1	2	8
2	**(PROTEIN)** Kacang tanah / polong / buncis / panjang / bendil / panggang, dhal, bijian, tofu, tempeh						1	2	8
3	Susu badan						1	2	8
4a	**(BAHAN TENUSU)** Susu formula;						1	2	8

	Jenama: ________<br><br>Jumlah dlm 24 jam: ____ oz X _____ kali								
4 b	**(BAHAN TENUSU)** Keju, yogurt, minumum yogurt						1	2	8
5 a	**(PROTEIN)** Daging lembu, khinzir kambing, ayam, itik						1	2	8
5 b	**(PROTEIN)** Organ dalaman - hati, ginjal, jantung						1	2	8
5 c	**(PROTEIN)** Ikan, makanan laut lain						1	2	8
6	**(PROTEIN)** Telur						1	2	8
7 a	**Sayur berwarna merah**: Carrot (lobak merah), kentang manis, labu						1	2	8
7 b	**Buah-buahan berwarna merah**: Mangga, betik, pisang, nenas, oren, nangka, chempedak						1	2	8

8a	**Sayur daun hijau**: Sawi, bayam, kailan, kangkong						1	2	8
8b	Buah-buahan atau sayur yang lain, timum, epel, terung						1	2	8
	Lain-lain								

		Pagi	Tengah pagi	Tengahari	Petang	Malam	Ya	Tidak	Tidak tahu
9a	**Minyak masakan:** Mentega, minyak zaiton/sapi, santan						1	2	8
9b	**Makanan diproses:** kentang goreng, sosej, nuget ayam						1	2	8
10	**Makanan bergula:** Coklat, gula-gula, kek, biskut						1	2	8
11	**Minuman bergula**: kordial buah-buahan, minuman ringan, minuman						1	2	8

	berkarbonasi, air sirap, *minuman* malt coklat								
12	**Makanan bungkusan**: biskut, mi segera, , makanan bayi						1	2	8
13	**Jus** buah-buahan yang **segar** diperah						1	2	8
14	Bumbu / perencah dan perasa seperti cili, rempah, herba, halia atau serbuk ikan.						1	2	8
16	Berapa kalikah anak anda makan makanan pejal, separa pejal atau lembut selain daripada cecair sepanjang hari semalam?	Jumlah kali [ ]<br><br>Tak pasti: [ ]							

17	Adakah anak anda mengambil mana-mana vitamin tambahan?	Ya [ ]; Sila nyatakn:________
18	Adakah makanan anak anda dikisar atau dihaluskan (puree)?	Ya [ ] Tidak [ ]<br><br>Jenis makanan: _______________
19	Adakah anak anada dalam kebiasaan ya disuap makan atau makan sendiri?	Disuap makan [ ] Makan sendiri [ ] Disuap makan dan makan sendiri [ ]
20	Adakah anak anda makan bersama dengan keluarga?	Ya [ ] Tidak [ ]

Tempoh penyusuan *susu* ibu secara eksklusif :

Penjaga utama seharian :

Tempoh masa skrin seharian :

Kaedah *storytelling* : Ya [ ] Tidak [ ]

Jangkamasa kandungan :

Berat lahir : ………………..gm; Panjang : ………………..cm; Lilitan kepala : ………………..cm

Berat semasa : ………………..gm; Panjang/ Tinggi semasa : ………………..cm; Lilitan kepala semasa : ………………..cm

Berat badan dan ketinggian ibu : ………………..kg; ………………..cm

Berat badan dan ketinggian bapa : ………………..kg; ………………..cm
